# Genome-wide comparative analysis of papain-like cysteine protease family genes in castor bean and physic nut

**DOI:** 10.1038/s41598-017-18760-6

**Published:** 2018-01-10

**Authors:** Zhi Zou, Qixing Huang, Guishui Xie, Lifu Yang

**Affiliations:** 1 Key Laboratory of Biology and Genetic Resources of Rubber Tree, Ministry of Agriculture, Rubber Research Institute, Chinese Academy of Tropical Agricultural Sciences, Baodaoxincun, Danzhou, 571737 Hainan Province China; 2 Institute of Tropical Biosciences and Biotechnology, Chinese Academy of Tropical Agricultural Sciences, Xueyuan Road 4, Haikou, 570100 Hainan Province China

## Abstract

Papain-like cysteine proteases (PLCPs) are a class of proteolytic enzymes involved in many plant processes. Compared with the extensive research in *Arabidopsis thaliana*, little is known in castor bean (*Ricinus communis*) and physic nut (*Jatropha curcas*), two Euphorbiaceous plants without any recent whole-genome duplication. In this study, a total of 26 or 23 PLCP genes were identified from the genomes of castor bean and physic nut respectively, which can be divided into nine subfamilies based on the phylogenetic analysis: RD21, CEP, XCP, XBCP3, THI, SAG12, RD19, ALP and CTB. Although most of them harbor orthologs in *Arabidopsis*, several members in subfamilies RD21, CEP, XBCP3 and SAG12 form new groups or subgroups as observed in other species, suggesting specific gene loss occurred in *Arabidopsis*. Recent gene duplicates were also identified in these two species, but they are limited to the SAG12 subfamily and were all derived from local duplication. Expression profiling revealed diverse patterns of different family members over various tissues. Furthermore, the evolution characteristics of PLCP genes were also compared and discussed. Our findings provide a useful reference to characterize PLCP genes and investigate the family evolution in Euphorbiaceae and species beyond.

## Introduction

Castor bean (*Ricinus communis* L., *2n* = 20) and physic nut (*Jatropha curcas* L., *2n* = 22) are two economically important species that belong to the spurge family, Euphorbiaceae^1,2^. Castor bean, a perennial shrub of the monotypic *Ricinus* genus, is indigenous to Africa. The oil produced in castor seeds, mainly composed of the unusual hydroxylated fatty acid ricinoleic acid, is widely used for industrial, medicinal and cosmetic purposes, having prompted its domestication in many tropical, subtropical and warm temperate regions around the world^3^. Physic nut, also known Barbados nut, purging nut or jatropha, is a semi-evergreen shrub or small tree originated from central America, and now is widely cultivated in tropical and subtropical regions around the world^4^. Physic nut is a potential energy plant with the high oil content (up to 50%) in seeds and the fossil fuel-like oil composition that can be easily processed into bio-diesel^5^. The draft genome of castor bean was reported in 2010, which consists of 25,878 scaffolds spanning approximate 400 Mb^6^. The genome size of physic nut was estimated to be 350 Mb and two genome assemblies have been available^7,8^. The more complete assembly is about 320 Mb consisting of 23,125 scaffolds, and the number of putative protein-encoding genes of 27,172 is slightly smaller than 31,221 in castor bean^6,8^. Most importantly, except for the ancient so-called γ whole-genome duplication (WGD) event shared by core eudicots including *Arabidopsis thaliana* and poplar (*Populus trichocarpa*), comparative genomics analysis indicated that both castor bean and physic nut didn’t experience additional recent WGD^6,8,9^. From this perspective, analysis of certain gene families in castor bean and physic nut may provide insights into lineage-specific evolution in high plants especially in core eudicots.

Papain-like cysteine proteases (PLCPs) are a class of proteolytic enzymes that are associated with plant growth, development, protein storage and mobilization, organ senescence, abscission, seed germination, immunity and stress response^10–14^. Featuring a catalytic cysteine as a nucleophile during proteolysis, PLCPs are classed as the family C1A of clan CA and thus are also known as C1A cysteine proteases^15^. PLCPs are produced as preproproteases which usually include a signal peptide, an auto-inhibitory pro-domain and a mature protease domain^16^. The signal peptide ensures that the proprotease enters the endomembrane system, whereas the pro-domain prevents premature activation of the protease. Thereby, the protease precursors are usually inactive or weakly active. To become active, PLCPs need to be processed either by self-processing or with the aid of processing enzymes, which depends on the pH, the action of other proteases and protease inhibitors, and the cellular or extracellular environment^10,17^. In animals, PLCPs are often called cathepsins, and PLCPs in plants are named cathepsin L-, B-, H-, or F-like based on sequence similarity. Furthermore, the L-like can be subclassed into five phylogenetic subgroups (i.e. A–E)^18^. Although several properties of individual PLCPs have been reported in a wide range of plant species^19–21^, the genome-wide analysis is still limited to several species such as *Arabidopsis*, poplar and rubber (*Hevea brasiliensis*) which were proven to have undergone two or one recent doubling events respectively^2,16,22–25^. In *Arabidopsis*, 31 PLCPs were recently divided into nine subfamilies based on the phylogenetic analysis^17^: Subfamily 9 (CTB3-like or CTB) contains three cathepsin B-like PLCPs; Subfamily 8 (ALP-like or ALP) contains two cathepsin H-like PLCPs with the vacuolar-targeting NPIR motif at the N-terminal; Subfamily 7 (RD19A-like or RD19) contains four cathepsin F-like PLCPs; Subfamily 6 (SAG12-like or SAG12) contains six members; both Subfamily 5 (THI1-like or THI) and Subfamily 4 (XBCP3-like or XBCP3) contain a single member; Subfamily 3 (XCP2-like or XCP) contains two members; Subfamily 2 (CEP1-like or CEP) contains three members with the endoplasmic reticulum (ER)-localizing KDEL motif at the C-terminal; Subfamily 1 (RD21A-like or RD21) contains nine members (see Supplementary Table S1).

In this study, genome-wide identification of castor bean and physic nut PLCP family genes was carried out. Their gene structures were manually curated through aligning transcriptome data to the gene-encoding scaffolds. Furthermore, the sequence feature, evolutionary relationship and expression pattern were also investigated and compared.

## Results

### Characterization of 26 PLCP genes in castor bean

The initial search of the castor bean genome resulted in 28 loci putatively encoding PLCP homologs. All of them were predicted by the genome annotation^6^, however, two loci (i.e. 29900.t000066 and 29675.t000004) were shown to encode pseudogenes and were not further analyzed. The remaining 26 loci are distributed across 16 scaffolds. Most scaffolds were found to harbor a single PLCP gene, however, six of them were shown to contain more than one, i.e., scaffold30170 (4), scaffold29646 (3), scaffold29900 (3), scaffold30131 (2), scaffold28962 (2) and scaffold29910 (2) (Table 1).

**Table 1:** List of 26 RcPLCP genes identified in this study.

Gene name	Locus ID	Protein ID	Scaffold	Predicted position	Identified position	EST hits	AS^a^	AA	MW (KDa)	*pI*	GRAVY	iPSORT^b^	At_ortholog^c^	Jc_ortholog^c^
*RcRD21A*	30170.t000243	30170.m013831	scaffold30170	1214556–1211809	1214722–1211316	16	Yes	469	52.11	5.39	−0.492	S	AtRD21A	JcRD21A
*RcRD21B*	29801.t000069	29801.m003124	scaffold29801	415594–412915	415851–412454	10	—	471	52.18	5.71	−0.470	S	—	JcRD21B
*RcRD21C*	29970.t000002	29970.m000973	scaffold29970	17319–21021	17149–21809	0	—	383	42.96	6.56	−0.419	M	AtRDL1	JcRD21C
*RcCEP1*	30147.t000097	30147.m013826	scaffold30147	2830277–2828642	2830593–2828056	3	Yes	360	40.11	5.97	−0.571	S	AtCEP1	JcCEP1
*RcCEP2*	29929.t000288	29929.m004785	scaffold29929	1643832–1642090	1643874–1641686	0	Yes	359	40.39	5.87	−0.603	S	—	JcCEP2
*RcXCP1*	30162.t000046	30162.m001301	scaffold30162	1780890–1779633	1781121–1779118	0	—	349	39.01	5.44	−0.390	S	AtXCP1	JcXCP1
*RcXCP2*	30170.t000213	30170.m013801	scaffold30170	4386293–4387733	4386238–4387922	1	—	349	39.03	5.25	−0.412	S	AtXCP2	JcXCP2
*RcXBCP3*	30170.t000524	30170.m014112	scaffold30170	2901779–2899595	2901979–2898207	1	—	466	51.59	7.43	−0.296	S	AtXBCP3	JcXBCP3
*RcXBCP3L*	29381.t000001	29381.m000072	scaffold29381	7721–11783	7678–12160	13	—	501	55.85	5.05	−0.432	S	—	JcXBCP3L
*RcTHI1*	29646.t000057	29646.m001109	scaffold29646	344102–345635	343762–345957	1	Yes	347	38.63	5.48	−0.439	S	AtTHI1	JcTHI1
*RcSAG12H1*	30131.t000408	30131.m007257	scaffold30131	2504598–2505766	2504200–2506430	0	—	362	41.11	6.17	−0.412	S	AtSAG12	JcSAG12H3
*RcSAG12H2*	28962.t000017	28962.m000448	scaffold28962	92991–94101	92944–94101	0	—	340	37.20	5.22	−0.441	S	AtSAG12	JcSAG12H4
*RcSAG12H3*	28962.t000018	28962.m000449	scaffold28962	96412–97522	96181–97735	0	Yes	340	37.46	5.16	−0.424	S	AtSAG12	JcSAG12H4
*RcSAG12H4*	29646.t000033	29646.m001085	scaffold29646	207629–208856	207526–209015	0	—	349	38.55	9.33	−0.347	S	AtSAG12	—
*RcSAG12H5*	29646.t000034	29646.m001086	scaffold29646	211653–212894	211496–213036	0	—	342	38.06	8.59	−0.417	S	AtSAG12	—
*RcSAG12H6*	29900.t000065	29900.m001603	scaffold29900	407069–405926	407069–405639	0	—	344	38.12	5.13	−0.428	S	AtSAG12	JcSAG12H7
*RcSAG12H7*	29910.t000015	29910.m000924	scaffold29910	208698–206791	208876–206709	0	—	341	37.41	4.86	−0.458	S	AtSAG12	JcSAG12H8
*RcSAG12H8*	29910.t000014	29910.m000923	scaffold29910	204533–202640	204533–202640	0	—	342	37.40	4.71	−0.439	S	AtSAG12	JcSAG12H8
*RcPAP1*	29900.t000078	29900.m001616	scaffold29900	487805–488928	487718–489114	0	—	340	37.64	6.33	−0.325	S	AtPAP1	—
*RcPAP2*	29900.t000077	29900.m001615	scaffold29900	483849–485660	483819–485847	1	—	343	37.89	4.87	−0.343	S	AtPAP1	—
*RcPAP3*	29827.t000145	29827.m002672	scaffold29827	836744–835493	836744–835443	0	—	342	37.91	5.07	−0.371	M	AtPAP1	—
*RcRD19A*	30131.t000249	30131.m007098	scaffold30131	1514779–1516461	1514766–1516903	8	Yes	373	41.07	5.95	−0.312	S	AtRD19A	JcRD19A
*RcRD19B*	30170.t000534	30170.m014122	scaffold30170	2964658–2966204	2964578–2966528	68	Yes	366	40.38	5.68	−0.249	S	AtRD19C	JcRD19B
*RcRD19C*	28462.t000004	28462.m000130	scaffold28462	46401–48802	46261–49312	17	Yes	381	41.73	5.87	−0.037	S	AtRD19D	JcRD19C
*RcALP1*	29739.t000193	29739.m003757	scaffold29739	1197717–1200174	1197581–1200666	3	Yes	358	39.21	5.88	−0.185	S	AtALP	JcALP1
*RcCTB1*	30076.t000074	30076.m004510	scaffold30076	399770–402553	399629–403162	2	Yes	359	39.84	5.90	−0.200	S	AtCTB2	JcCTB1

^a^“Yes” represents genes containing alternative splicing isoforms; ^b^“S, M and C” represent signal peptide, mitochondrial targeting peptide or chloroplast transit peptide, respectively; ^c^The best ortholog hit.

Except for *RcCEP1* (GenBank accession number AF050756)^26^, homology analysis showed that no full-length cDNA sequences of the other 25 RcPLCP genes were reported in any public database (as of Dec 2016). Nevertheless, 13 members had EST (expressed sequence tag) hits in GenBank and *RcRD19B* was found to harbor the maximal hit of 68 ESTs. Moreover, the expression of other genes was supported by RNA sequencing reads derived from various tissue transcriptomes, i.e. leaf, flower, endosperm and seed^27–29^. Except for *RcSAG12H8*, the transcription regions of all other RcPLCP genes were successfully extended based on the read alignment (Table 1).

Since the gene models released in castor bean were the result of an automatic annotation^6^, an expert revision of their gene structures was conducted *via* mapping ESTs and RNA sequencing reads to the identified scaffolds. Interesting, six out of the 26 annotated gene models were proved to be inaccurate. The locus 29970.t000002 (*RcRD21C*) was predicted to encode 366 residues (29970.m000973), and it represents only the 3′ sequence of the gene which encodes 383 residues (see Supplementary File S1). The locus 30162.t000046 (*RcXCP1*) was predicted to contain three introns putatively encoding 324 residues (30162.m001301), however, hundreds of RNA sequencing reads indicated that the “GAAA” sequence in the first exon was absent from the genome assembly. Thereby, this locus promises to harbor two introns encoding 349 residues (see Supplementary File S2). The locus 30170.t000524 (*RcXBCP3*) was predicted to contain four introns putatively encoding 422 residues (30170.m014112), however, read alignment indicated that this locus harbors five introns putatively encoding 466 residues (see Supplementary File S3). The locus 29381.t000001 (*RcXBCP3L*) was predicted to encode 417 residues (29381.m000072), however, read alignment indicated that this locus encodes 501 residues (see Supplementary File S4). The locus 29827.t000145 (*RcPAP3*) was predicted to contain two introns putatively encoding 321 residues (29827.m002672), however, read alignment indicated that this locus harbors one intron putatively encoding 342 residues (see Supplementary File S5). The locus 30076.t000074 (*RcCTB1*) was predicted to encode 376 residues (30076.m004510), however, read alignment indicated that this locus encodes 359 residues (see Supplementary File S6). Additionally, 10 genes (i.e. *RcRD21A*, *RcCEP1*, *RcCEP2*, *RcTHI1*, *RcSAG12H3*, *RcRD19A*, *RcRD19B*, *RcRD19C*, *RcALP1* and *RcCTB1*) were shown to have alternative splicing isoforms (Table 1).

Four gene pairs (i.e. paralogs) can be defined as tandem duplicates for their close organization on same scaffolds and high sequence identity, i.e., 97.4% between *RcSAG12H*7 (29910.t000015) and *RcSAG12H*8 (29910.t000014), 96.8% between *RcSAG12H2* (28962.t000017) and *RcSAG12H3* (28962.t000018), 87.5% between *RcSAG12H4* (29646.t000033) and *RcSAG12H5* (29646.t000034), and 74.1% between *RcPAP1* (29900.t000078) and *RcPAP2* (29900.t000077). However, whether *RcPAP3* is a proximal duplicate of *RcPAP1* or *RcPAP2* still needs to be confirmed, since the 25,878 assembled scaffolds have not been anchored to the chromosomes yet^6^.

### Characterization of 23 PLCP genes in physic nut

**Figure 1:**
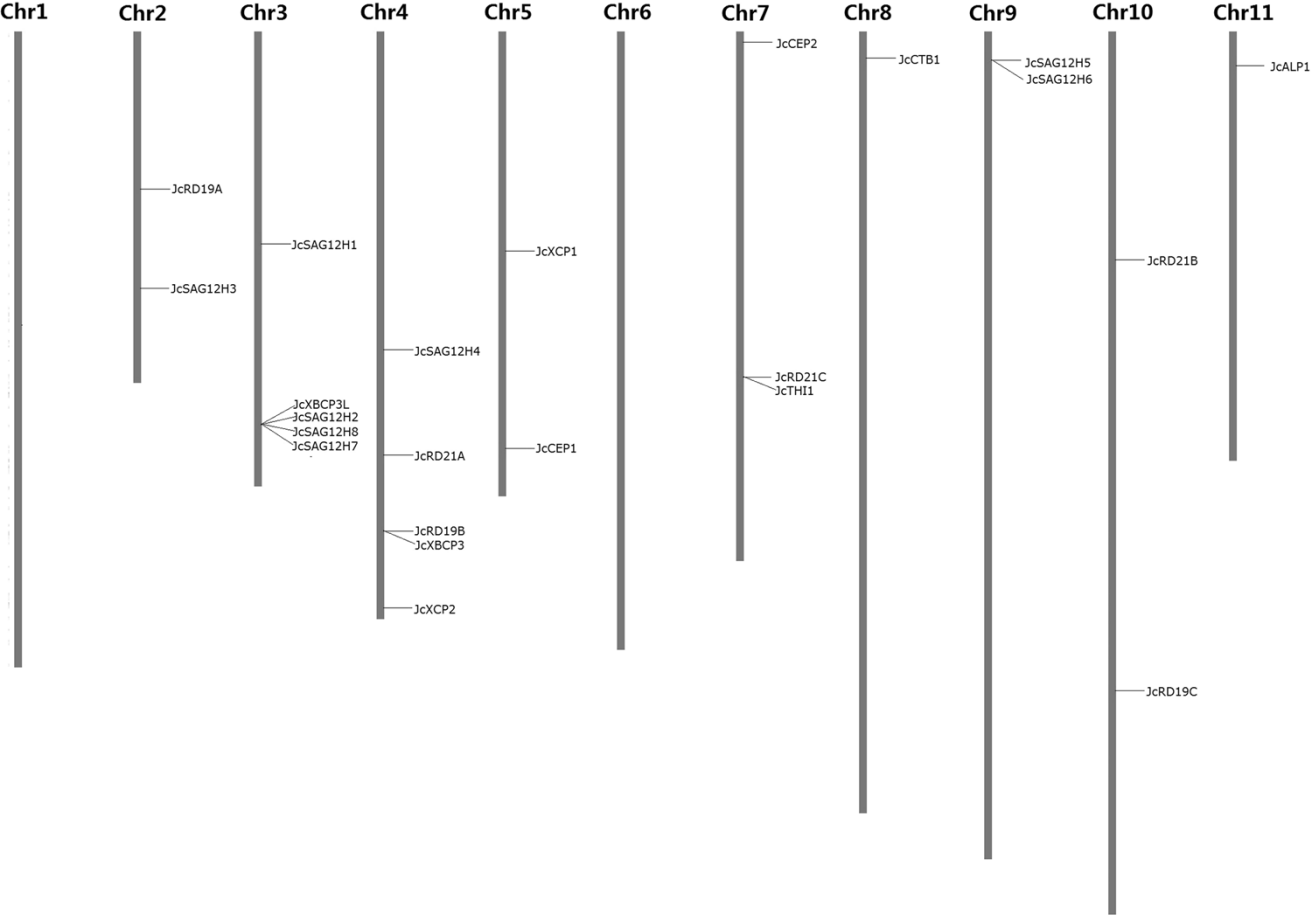
Chromosomal distribution of 23 JcPLCP genes. The eleven linkage groups or chromosomes were constructed with 1208 DNA markers, where the chromosome number is indicated at the top.

After discarding four pseudogenes (i.e. JCGZ_22119, JCGZ_05109 and two unpredicted loci on scaffold170), a total of 23 PLCP-encoding loci were identified from the physic nut genome. Among them, 22 loci were predicted by the automatic genome annotation^8^, whereas one more locus putatively encoding a SAG12 subfamily member was identified from scaffold684 (i.e. *JcSAG12H8*, see Supplementary File S7). These loci are distributed across 17 scaffolds. Like observed in castor bean, most scaffolds contain one PLCP gene, and four scaffolds encoding more than one are as follows: scaffold684 (4), scaffold341 (2), scaffold159 (2) and scaffold872 (2) (Table 2). When taking the linkage map with 1208 markers^8^ into account, these scaffolds can be further anchored to nine chromosomes (Chrs), i.e., Chr2 (scaffold84 and scaffold96), Chr3 (scaffold26 and scaffold684), Chr4 (scaffold5, scaffold46, scaffold159 and scaffold221), Chr5 (scaffold3 and scaffold328), Chr7 (scaffold341 and scaffold502), Chr8 (scaffold392), Chr9 (scaffold872), Chr10 (scaffold7 and scaffold464) and Chr11 (scaffold211). The distribution of JcPLCP genes looks uneven: Chromosomes 3 and 4 contain the most of five PLCP genes, followed by Chromosome 7 with three genes (Fig. 1).

**Table 2:** List of 23 JcPLCP genes identified in this study.

Gene name	Locus ID	Scaffold	Predicted position	Identified position	Chr	EST hits	AS^a^	AA	MW (KDa)	*pI*	GRAVY	iPSORT^b^	At_ortholog^c^	Rc_ortholog^c^
*JcRD21A*	JCGZ_16099	scaffold46	3722788–3725887	3726134–3722381	4	53	—	466	51.79	5.28	−0.515	S	AtRD21A	RcRD21A
*JcRD21B*	JCGZ_22120	scaffold7	104053–105002	103886–106508	10	6	—	475	52.68	5.39	−0.504	S	—	RcRD21B
*JcRD21C*	JCGZ_12447	scaffold341	1511831–1514897	1511754–1515287	7	3	Yes	366	41.12	5.40	−0.372	S	AtRDL1	RcRD21C
*JcCEP1*	JCGZ_11373	scaffold328	482830–484642	484817–482666	5	30	—	360	40.17	5.69	−0.552	S	AtCEP1	RcCEP1
*JcCEP2*	JCGZ_17869	scaffold502	2577993–2579143	2579634–2577807	7	36	—	358	39.94	6.19	−0.548	S	—	RcCEP2
*JcXCP1*	JCGZ_10746	scaffold3	258651–259875	258620–260019	5	0	Yes	349	39.05	5.59	−0.361	S	AtXCP1	RcXCP1
*JcXCP2*	JCGZ_08041	scaffold221	310092–311414	311442–309951	4	0	—	350	39.18	5.40	−0.367	S	AtXCP2	RcXCP2
*JcXBCP3*	JCGZ_04495	scaffold159	1388654–1391224	1391430–1388201	4	0	Yes	441	48.82	6.06	−0.333	S	AtXBCP3	RcXBCP3
*JcXBCP3L*	JCGZ_21572	scaffold684	2356094–2358380	2355980–2358669	3	3	—	524	58.04	5.24	−0.369	C	—	RcXBCP3L
*JcTHI1*	JCGZ_12228	scaffold341	82305–83446	83709–81708	7	6	Yes	347	38.94	8.43	−0.615	S	AtTHI1	RcTHI1
*JcSAG12H1*	JCGZ_09604	scaffold26	600732–601907	602051–600546	3	0	—	345	38.26	7.99	−0.438	S	AtSAG12	—
*JcSAG12H2*	JCGZ_21557	scaffold684	2264217–2264672	2264714–2263227	3	0	—	345	38.40	8.56	−0.377	S	AtSAG12	—
*JcSAG12H3*	JCGZ_24483	scaffold84	187704–188806	189021–187389	2	0	—	339	37.60	5.94	−0.424	S	AtSAG12	RcSAG12H1
*JcSAG12H4*	JCGZ_17185	scaffold5	199542–200754	199432–201896	4	0	—	340	37.42	5.13	−0.413	S	AtSAG12	RcSAG12H2
*JcSAG12H5*	JCGZ_25371	scaffold872	469319–470468	472278–473649	9	0	—	340	37.59	5.01	−0.424	S	AtSAG12	RcSAG12H2
*JcSAG12H6*	JCGZ_25372	scaffold872	472278–473548	469201–470468	9	0	—	340	37.28	6.90	−0.426	S	AtSAG12	RcSAG12H2
*JcSAG12H7*	JCGZ_21549	scaffold684	2211946–2213074	2213483–2211423	3	0	—	344	37.98	4.73	−0.372	S	AtSAG12	RcSAG12H6
*JcSAG12H8*	—	scaffold684	—	2215711–2214317	3	0	—	342	37.48	4.97	−0.413	S	AtSAG12	RcSAG12H7
*JcRD19A*	JCGZ_26761	scaffold96	231461–233265	233346–230988	2	67	—	370	40.56	5.95	−0.246	S	AtRD19A	RcRD19A
*JcRD19B*	JCGZ_04503	scaffold159	1432232–1434263	1432160–1434615	4	11	—	368	40.92	5.81	−0.338	S	AtRD19C	RcRD19B
*JcRD19C*	JCGZ_16165	scaffold464	314963–316717	316756–314735	10	11	—	383	42.10	6.03	−0.106	S	AtRD19D	RcRD19C
*JcALP1*	JCGZ_07488	scaffold211	2991934–2994769	2994891–2991702	11	51	Yes	358	39.44	5.88	−0.147	S	AtALP	RcALP1
*JcCTB1*	JCGZ_14145	scaffold392	1649834–1653016	1653334–1649409	8	7	—	358	39.71	6.07	−0.230	S	AtCTB2	RcCTB1

^a^“Yes” represents genes containing alternative splicing isoforms; ^b^“S, M and C” represent signal peptide, mitochondrial targeting peptide or chloroplast transit peptide, respectively; ^c^The best ortholog hit.

As of Dec 2016, although no full-length cDNA sequences were available for all physic nut PLCP family genes, 12 members were found to have EST hits in GenBank and *JcRD19A* harbored the maximum of 67 hits. Moreover, the expression of other members was supported by RNA sequencing reads derived from transcriptomes of various tissues including callus, root, leaf, inflorescence meristem, flower, embryo and seed^7,8,30–34^. Based on the read alignment, the transcription regions of all JcPLCP genes were extended and seven predicted gene models were optimized (Table 2). The locus JCGZ_22120 (*JcRD21B*) was predicted to encode 242 residues, and it represents only the 5′ sequence of the gene which encodes 471 residues (see Supplementary File S8). The locus JCGZ_17869 (*JcCEP2*) was predicted to encode 269 residues, and it represents only the 3′ sequence of the gene which encodes 358 residues (see Supplementary File S9). The locus JCGZ_21572 (*JcXBCP3L*) was predicted to encode 508 residues, and it represents only the 3′ sequence of the gene which encodes 524 residues (see Supplementary File S10). The locus JCGZ_12228 (*JcTHI1*) was predicted to encode 315 residues, and it represents only the 3′ sequence of the gene which encodes 347 residues (see Supplementary File S11). The locus JCGZ_09604 (*JcSAG12H1*) was predicted to encode 311 residues, and it represents only the 3′ sequence of the gene which encodes 345 residues (see Supplementary File S12). The locus JCGZ_21557 (*JcSAG12H2*) was predicted to encode 155 residues, and it represents only the 5′ sequence of the gene which encodes 345 residues (see Supplementary File S13). The locus JCGZ_21549 (*JcSAG12H7*) was predicted to encode 324 residues, and it represents only the 3′ sequence of the gene which encodes 344 residues (see Supplementary File S14). Additionally, five genes (i.e. *JcRD21C*, *JcXCP1*, *JcXBCP3*, *JcTHI1* and *JcALP1*) were shown to have alternative splicing isoforms (Table 2).

Several gene pairs were shown to exhibit high sequence identity, i.e., 97.8% between *JcSAG12H1* and *JcSAG12H2*, 88.6% between *JcSAG12H5* and *JcSAG12H6*, 76.4% between *JcSAG12H7* and *JcSAG12H8*, 67.2% between *JcSAG12H8* and *JcSAG12H1*, 66.8% between *JcSAG12H8* and *JcSAG12H2*, 65.4% between *JcSAG12H7* and *JcSAG12H1*, 65.2% between *JcSAG12H7* and *JcSAG12H2*. *JcSAG12H2*/*7*/*8* and *JcSAG12H5*/6 can be defined as tandem duplicates for their adjacent organization on same scaffolds, whereas *JcSAG12H1* can be defined as the recent proximal duplicate of *JcSAG12H2* for their distribution on two distinct scaffolds of Chromosome 3 (Table 2 and Fig. 1).

### Phylogenetic analysis of castor bean and physic nut PLCPs

**Figure 2:**
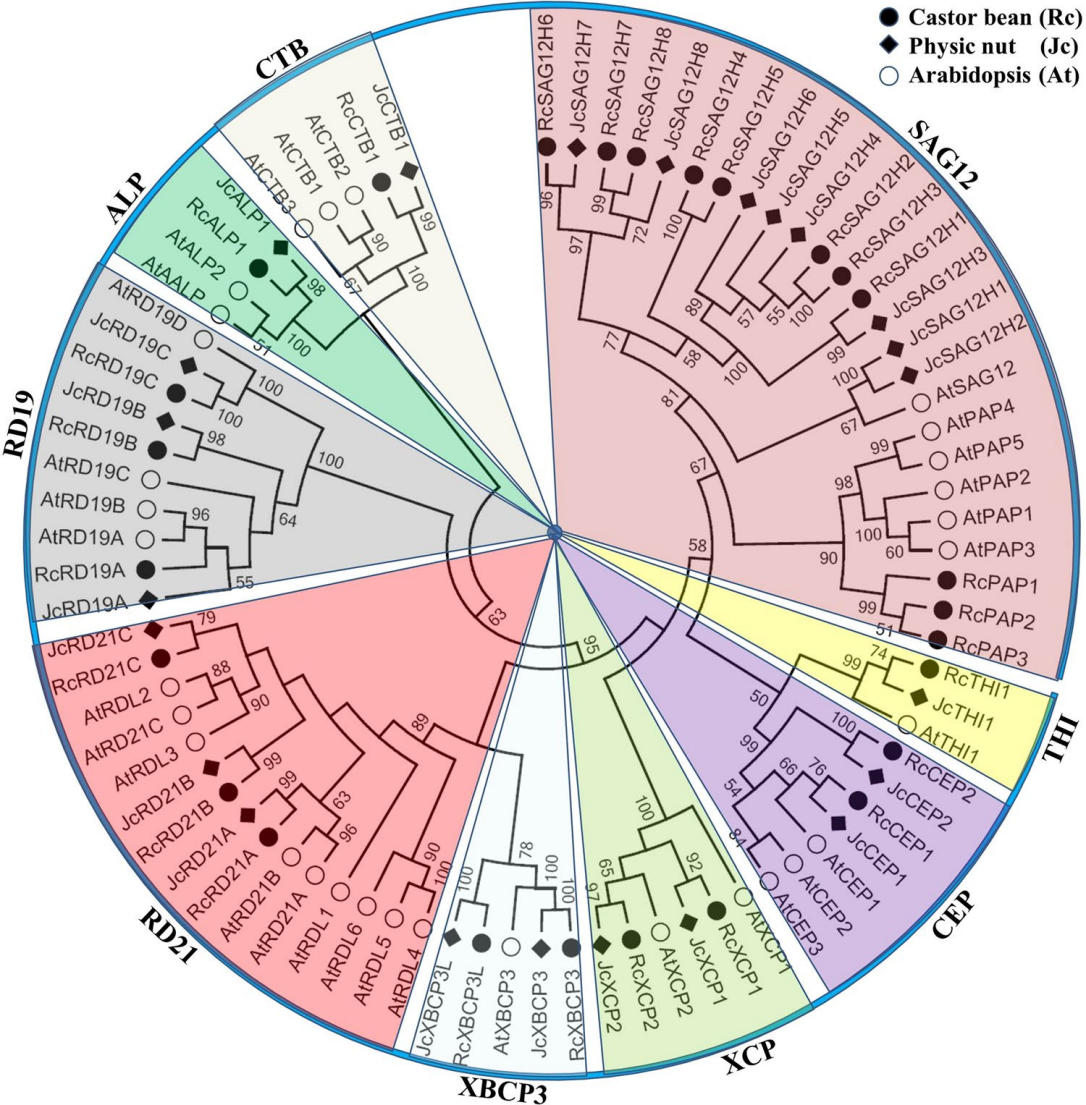
Phylogenetic analysis of castor bean, physic nut and *Arabidopsis* PLCPs. Sequence alignment and construction of the phylogenetic tree were performed using MUSCLE or MEGA6, respectively. The subfamily names are indicated next to their cluster and the distance scale denotes the number of amino acid substitutions per site.

**Figure 3:**
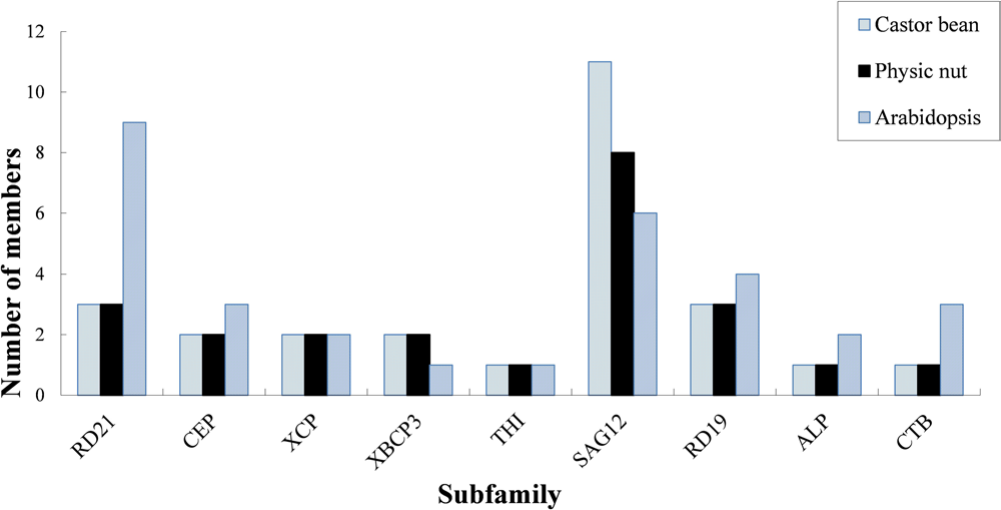
Distribution of castor, physic nut and *Arabidopsis* PLCP genes in subfamilies.

According to the reciprocal BLASTP analysis, 26 RcPLCPs have 20 or 14 orthologous groups (OGs) in physic nut and *Arabidopsis*, respectively (Table 1), and 23 JcPLCPs have 19 or 13 OGs in castor bean and *Arabidopsis*, respectively (Table 2), suggesting gene-specific duplication and/or loss occurred. To reveal the evolutionary relationship, we constructed a phylogenetic tree using 80 PLCPs from castor bean, physic nut as well as the extensively studied *Arabidopsis*. Results showed that castor bean and physic nut PLCPs can be divided into nine subfamilies as described in *Arabidopsis*, i.e., RD21, CEP, XCP, XBCP3, THI, SAG12, RD19, ALP and CTB (Figs 2 and 3). Furthermore, RcRD21B/JcRD21B, RcCEP2/JcCEP2 and two XBCP3 members (named RcXBCP3L/JcXBCP3L), which have no orthologs in *Arabidopsis*, were found to form new groups (Tables 1 and 2, and Fig. 2).

As shown in Fig. 2, a large number of AtPLCPs were grouped in pairs, reflecting the occurrence of two recent WGD events^22^. These gene pairs are widely distributed in different subfamilies, only excluding subfamilies XBCP3 and THI with a single member. In contrast, few gene pairs were found in castor bean and physic nut, which are limited to the SAG12 subfamily. In *Arabidopsis*, the SAG12 subfamily is composed of six members, which can be further divided into two groups named SAG12 and PAP. The PAP group was also present in castor bean but absent from physic nut, suggesting specific gene loss in the latter. Compared with *Arabidopsis* that contains a single SAG12 group member, both castor bean and physic nut contain eight members, which were shown to form four subgroups (Fig. 2).

### Gene structure, sequence feature and conserved motifs

**Figure 4:**
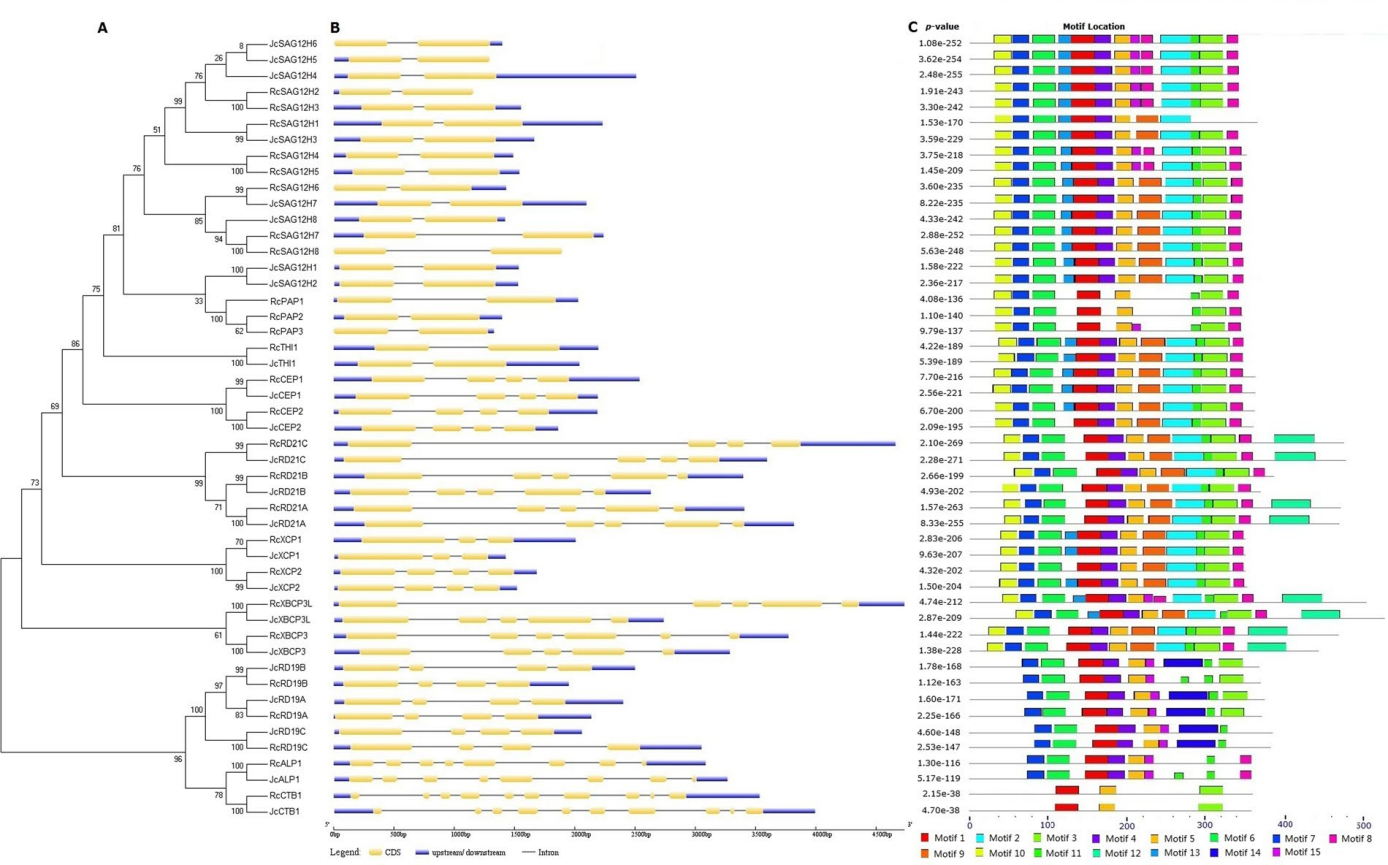
Structural features of castor and physic nut PLCP genes. (**A**) An unrooted phylogenetic tree constructed using MEGA6. (**B**) A graphic representation of exon-intron structures displayed using GSDS. (**C**) Distribution of 15 conserved motifs.

The exon-intron structure of castor bean and physic nut PLCP genes was investigated based on optimized gene models, which are supported by ESTs and/or RNA sequencing reads. As shown in Fig. 4B, these genes harbor at least one intron, varying from one to ten as observed in *Arabidopsis*. Although genes in different subfamilies were shown to harbor distinct exon-intron structures, the structure is usually conserved within the subfamily and between orthologs across three compared species. Without any exception, genes in the ALP subfamily all contain seven introns. Except for *AtSAG12* containing two introns, other members in subfamilies SAG12 and THI feature one intron. Most genes in subfamilies CEP, XCP and RD19 contain three introns, whereas *RcXCP1*, *JcXCP1*, *AtCEP1*, *AtCEP2*, *AtCEP3*, *AtRD19A* and *AtRD19B* contain two introns instead. Genes in the XBCP3 subfamily usually contain four introns, while *RcXBCP3* contains five introns instead. Genes in the CTB subfamily usually contain ten introns, however, *AtCTB1* harbors nine introns instead. Compared with the ORF (open reading frame) length (1023–1506 bp with the average of 1119 bp in castor bean, 1020–1575 bp with the average of 1135 bp in physic nut, and 1026–1392 bp with the average of 1118 bp in *Arabidopsis*), the gene size (from start to stop codons) of each gene is relatively more variant (1111–4315 bp with the average of 1933 bp in castor bean, 1103–3183 bp with the average of 1860 bp in physic nut, and 1137–2471 bp with the average of 1603 bp in *Arabidopsis*) **(**Fig. 4B and Supplementary Table S1).

Sequence analysis showed that the deduced PLCP proteins were predicted to harbor an average MW (molecular weight) of 41 kDa and *p*I (isoelectric point) value of 4.7. Since the predicted GRAVY (grand average of hydropathicity) values were all shown to be less than 0 (varying from −0.04 to −0.62), these proteins are more likely to be hydrophilic. According to the subcellular localization analysis, a hydrophobic signal peptide was also found at the N-terminal of each protein (Tables 1 and 2, and Supplementary Table S1), where JcALP1 and RcALP1 include the NPIR motif for the vacuolar localization as observed in AtALP^35^. Except for JcCEP2 that harbors a RDEL motif at the C-terminal, RcCEP1, RcCEP2 and JcCEP1 contain a KDEL motif for the ER retention^26,36,37^.

Motif compositions among different Rc/JcPLCPs were also investigated and the results were shown in Fig. 3 and Supplementary Fig. S1. Among the 15 motifs identified using MEME, Motifs 1–11 and 13 are broadly distributed. Motif 7 includes the ERFNIN consensus sequence. This motif as well as Motifs 10 and 6 are characterized as the well-studied Inhibitor_I29 (PF08246), which is the core of the auto-inhibitory pro-domain^10,16^. Motifs 1, 4, 5, 9, 15, 2, 11, 3 and 8 are characterized as the Peptidase_C1 domain (PF00112), where Motifs 1, 11 and 3 include the Cys, His or Asn active site respectively^10,11^. Motifs 14 and 15 are also part of the Peptidase_C1 domain, where Motif 14 is specific to the RD19 subfamily and Motif 15 is only found in subfamilies SAG12, XBCP3, RD19 and ALP. Motif 13 is the link of the Inhibitor_I29 domain and the Peptidase_C1 domain, which was shown to contain the cleavage site for generation of a mature enzyme^21,38^. Motif 12, which is limited to RD21 and XBCP3 subfamilies, is characterized as the well-studied GRAN domain (PF00396) (Fig. 3C).

### Expression patterns of RcPLCP genes in various tissues

**Figure 5:**
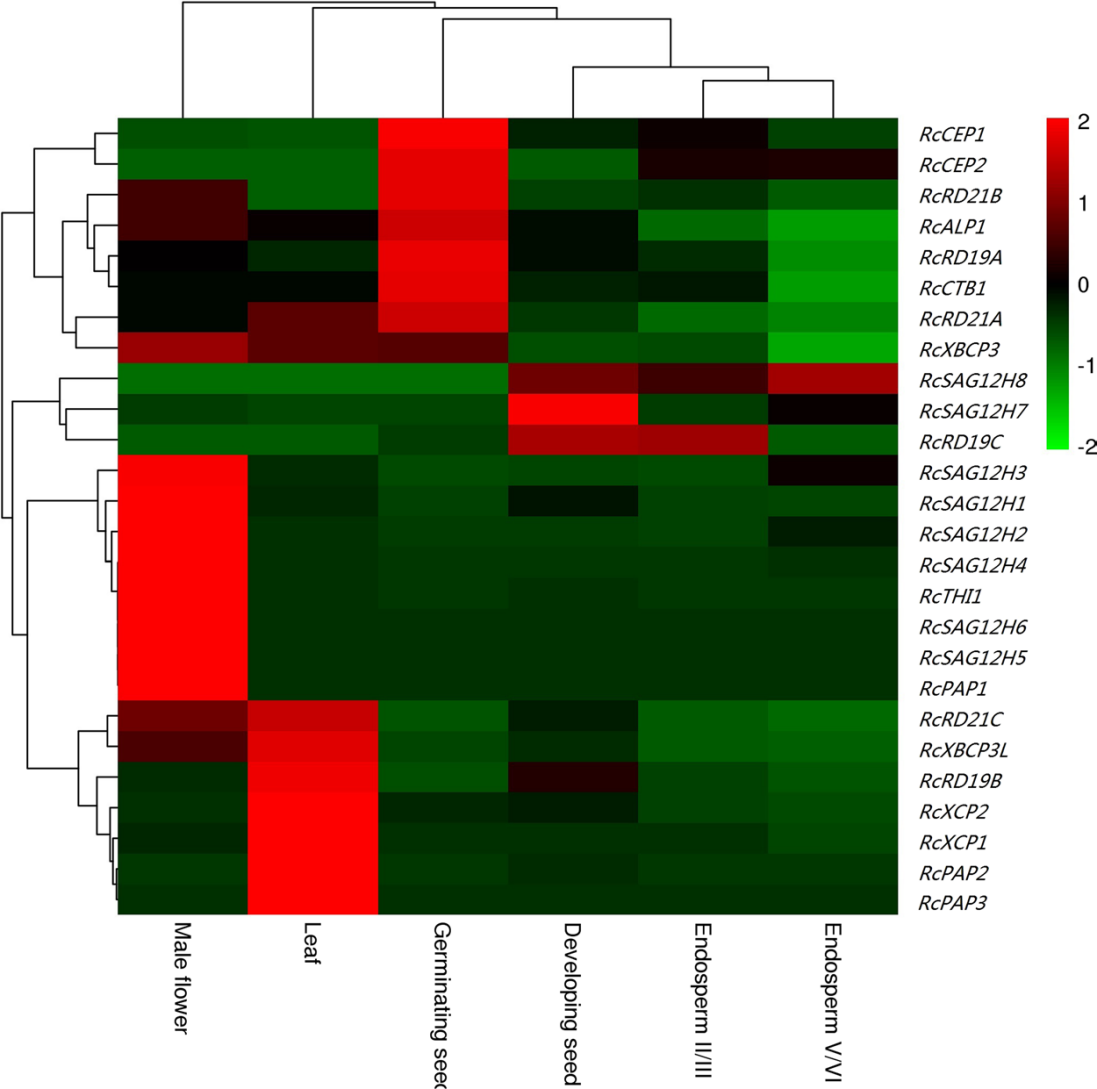
Transcriptional profiling of 26 RcPLCP genes over various tissues. Color scale denotes FPKM normalized log_10_ transformed counts.

Transcriptional profiling revealed that 26 RcPLCP genes were expressed in at least one of the tested tissues or developing stages of a certain tissue, i.e., 20 in leaf, 23 in male flower, 19 in endosperm II/III, 16 in endosperm V/VI, 21 in developing seed and 17 in germinating seed. According to the FPKM value, the total transcripts of RcPLCP genes were most abundant in male flower, followed by germinating seed (Group I); moderate in endosperm II/III, leaf and developing seed (Group II, occupying 21–27% of Group I); and, considerably low in endosperm V/VI (Group III, occupying 6–7% of Group I). In male flower, the unique member of the THI subfamily contributes the major transcripts, occupying about 72% of the total PLCP transcripts; by contrast, the second highly abundant RD21 subfamily occupies only 10%. In leaf, subfamilies RD19 and RD21 occupy about 69% of the total PLCP transcripts. In endosperm and seed, the CEP subfamily usually contributes the major PLCP transcripts, though the RD19 subfamily plays a more important role in developing seed. In endosperm V/VI, the CEP subfamily occupies about 88% of the total PLCP transcripts. In endosperm II/III, the CEP subfamily occupies about 57% of the total PLCP transcripts, and the less abundant subfamilies RD19 and RD21 occupy about 19% or 13% respectively. In germinating seed, the CEP subfamily occupies about 51% of the total PLCP transcripts, and the less abundant subfamilies RD21 and RD19 occupy about 25% or 11% respectively. In developing seed, three highly abundant subfamilies RD19, CEP and RD21 occupy about 35%, 28% or 17% of the total PLCP transcripts respectively (Fig. 5).

Although the transcript level is diverse, most RcPLCP genes were shown to express in all examined tissues. *RcCEP1* represents the most expressed gene in endosperm II/III, endosperm V/VI, developing seed and germinating seed, occupying about 46%, 41%, 43% or 27% of the total PLCP transcripts in each sample respectively; by contrast, its transcript level in leaf is extremely low. Compared with *RcCEP1*, the transcript level of *RcCEP2* is comparable in endosperm V/VI, but is relatively lower in other tissues. *RcTHI1* represents the most expressed gene in flower; *RcRD19B* and *RcRD21A* represent the most expressed genes in leaf, occupying about 29% or 25% of the total PLCP transcripts respectively. Nevertheless, the expression of *RcRD19B* was not detected in endosperm V/VI; *RcSAG12H5*, *RcSAG12H6* and *RcPAP1* seem to be flower-specific; and, *RcPAP3* seems to be leaf-specific (Fig. 5).

Based on the expression pattern across various tissues, 26 RcPLCP genes were clustered into four groups: Group 1 prefers to express in germinating seed, including 2 CEPs (*RcCEP1* and *RcCEP2*), 2 RD21s (*RcRD21B* and *RcRD21A*), *RcRD19A*, *RcALP1*, *RcCTB1* and *RcXBCP3*; Group 2 prefers to express in developing seed and endosperm, including *RcRD19C*, 2 SAG12s (*RcSAG12H7* and *RcSAG12H8*); Group 3 prefers to express in male flower, including *RcTHI1*, 7 SAG12s (*RcSAG12H1*, *RcSAG12H2*, *RcSAG12H3*, *RcSAG12H4*, *RcSAG12H5*, *RcSAG12H6* and *RcPAP1*); and, Group 4 prefers to express in leaf, including 2 XCPs (*RcXCP1* and *RcXCP2*), 2 SAG12s (*RcPAP2* and *RcPAP3*), *RcRD21C*, *RcXBCP3L* and *RcRD19B*.

### Expression patterns of JcPLCP genes in various tissues

**Figure 6:**
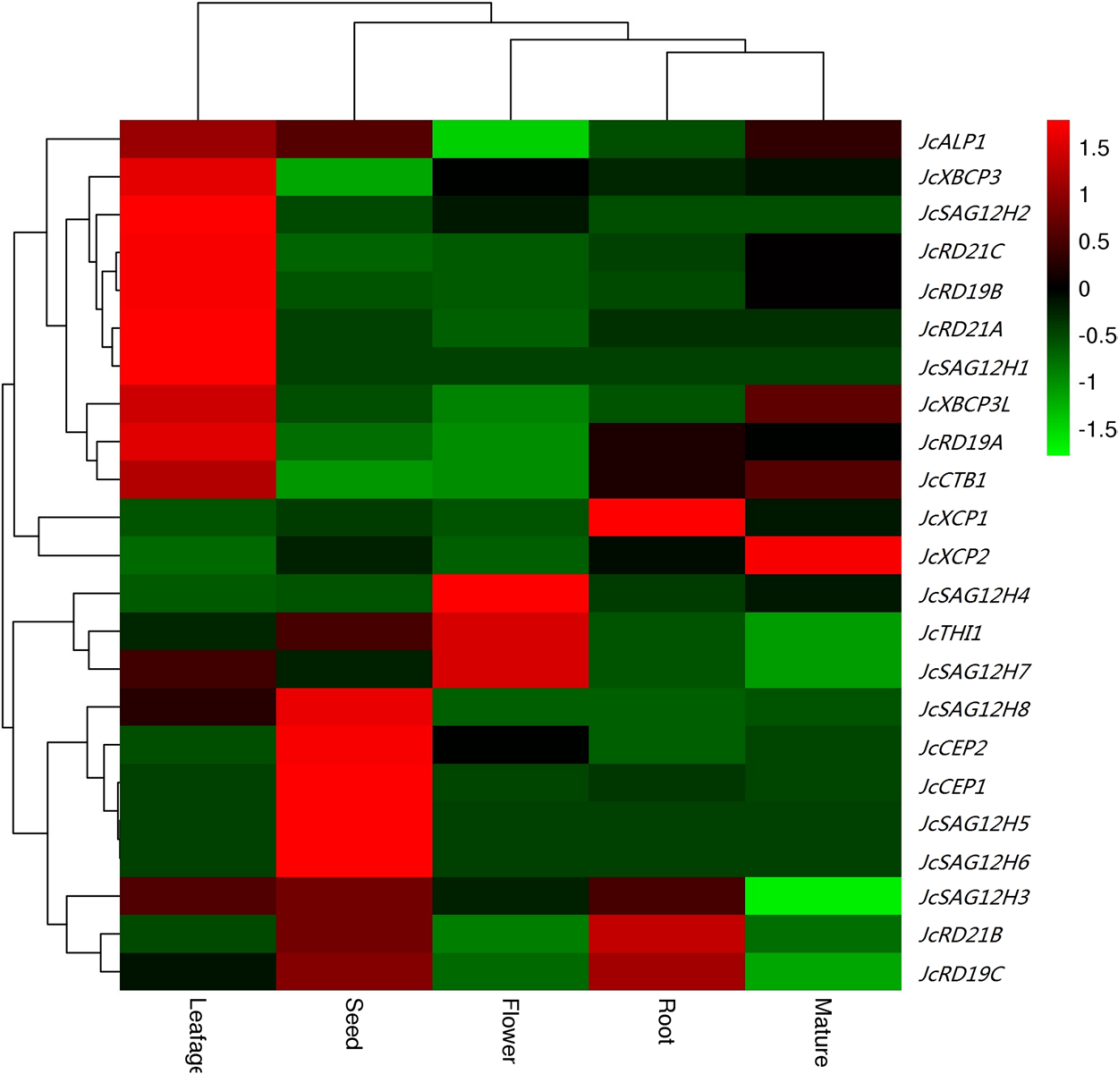
Transcriptional profiling of 23 JcPLCP genes over various tissues. Color scale denotes FPKM normalized log_10_ transformed counts.

As shown in Fig. 6, transcriptional profiling supported the expression of all JcPLCP genes in at least one of the examined tissues, i.e., 21 in root, 19 in flower bud, 23 in seed, 21 in leafage and 18 in mature leaf. The total transcripts were most abundant in leafage (Group I); moderate in seed, mature leaf and root (Group II, occupying 37–47% of Group I); and, considerably low in flower bud (Group III, occupying about 13% of Group I). In most tissues, RD19 and RD21 subfamilies contribute the major PLCP transcripts, e.g., 72% in leafage, 62% in flower, and 54% in root. By contrast, subfamilies RD19 and ALP occupy 55% of the total PLCP transcripts in mature leaf, and the CEP subfamily occupies 55% of the total PLCP transcripts in seed. Several important JcPLCP genes were also identified for a certain tissue. *JcRD19A* presents the most expressed gene in flower bud and root, both occupying about 30% of the total PLCP transcripts. *JcCEP1* presents the most expressed gene in seed, occupying about 55% of the total PLCP transcripts, though its expression level is relatively low in flower bud, leafage and mature leaf. In leafage, the transcript level of *JcRD21A*, *JcRD19A*, *JcRD19B* and *JcALP1* is considerably high, where *JcALP1* also presents the most expressed gene in mature leaf (Fig. 6). These genes were also clustered into four groups based on the tissue-specific expression pattern: Group 1 prefers to express in leafage, including 2 RD21s (*JcRD21A* and *RcRD21C*), 2 RD19s (*JcRD19A* and *RcRD19B*), 2 XBCP3 (*JcXBCP3* and *JcXBCP3L*), 2 SAG12 (*JcSAG12H1* and *JcSAG12H2*), *JcALP1* and *JcCTB1*; Group 2 prefers to express in root (*JcXCP1*) or mature leaf (*JcXCP2*); Group 3 prefers to express in flower bud, including *JcTHI1*, 2 SAG12s (*JcSAG12H4* and *JcSAG12H7*); and, Group 4 prefers to express in seed, including 2 CEP (*JcXCP1* and *JcXCP2*), 4 SAG12s (*JcSAG12H3*, *JcSAG12H5*, *JcSAG12H6*, and *JcSAG12H8*), *JcRD21B* and *JcRD19C*.

## Discussion

### Small number but high diversity of PLCP family genes in castor bean and physic nut

WGDs are widespread and play an important role in the origin and diversification of the angiosperms^39,40^. *Arabidopsis*, an annual herb with a relatively short life cycle and small size, services as a popular model species for the study of plant biology and genetics. The relatively small diploid genome (approximately 135 Mb) made it the first plant to be sequenced, completed in December of the year 2000^41^. However, analysis of the *Arabidopsis* genome has revealed several unexpected secrets. During the last 120 million years, *Arabidopsis* was shown to have experienced three WGDs known as γ, β and α^22,42^. The γ event occurred at about 117 million years ago (Mya)^9^, which is shared by all core eudicots, e.g. castor bean, physic nut, rubber, cassava (*Manihot esculenta*), poplar, papaya (*Carica papaya*), cacao (*Theobroma cacao*) and grapevine (*Vitis vinifera*)^6,8,24,25,43–47^. The β event occurred at 61–65 Mya, shortly after its divergence with its close species papaya^44^, which is shared by the Cleomaceae plants^48^. The α event is Brassicaceae-specific, occurred within a window of 23–50 Mya^22,49^. Following these WGD events, the ancestral *A. thaliana* genome was hugely rearranged and gene copies have been massively lost, and almost half of the genome was lost since its divergence with *A. lyrata* at about 10–13 Mya^50^. The genome-wide analysis indicated that the *Arabidopsis* PLCP gene family is comprised of 31 members that can be divided into nine subfamilies based on sequence similarity^17^. As shown in Fig. 2, a relatively high number of *Arabidopsis* PLCP gene pairs were identified in most subfamilies. The 18 duplicates were shown to be resulted from different modes of gene duplication, i.e., γ (2), β (2), α (5), tandem (5), proximal (1) and transposed (3)^51^ (see Supplementary Table S1).

Despite containing more or comparable protein-coding loci than *Arabidopsis* (i.e. 27,416 in TAIR10), our genome-wide survey revealed that castor bean (i.e. 31,221) and physic nut (i.e. 27,172) encode relatively less PLCP genes, i.e. 26 or 23, respectively. The number occupies 0.08% of the total loci in both species, which is relatively smaller than 0.11% in *Arabidopsis*. The expression of all these genes was supported by available EST and/or RNA sequencing reads, suggesting that they may have function in these two species. Moreover, all PLCP genes in physic nut can be anchored to nine out of the 11 chromosomes based on available DNA markers^8^. Based on the phylogenetic analysis, these genes can be assigned to nine previously described subfamilies (i.e. RD21, CEP, XCP, XBCP3, THI, SAG12, RD19, ALP and CTB)^17^.

Except for the SAG12 subfamily, one-to-one orthologous relationships were found between castor bean and physic nut, and conserved synteny between these two species can be clearly observed. Despite the castor bean genome is highly fragmented, we are able to anchor 25 out of 26 RcPLCP genes to eight physic nut chromosomes based on the synteny analysis (Supplementary Fig. S2). By contrast, gene-specific expansion was observed in the SAG12 subfamily, which can be further divided into two groups (i.e., SAG12 and PAP). The PAP group was shown to be lost in physic nut, but highly expanded in castor bean (i.e. 3 members) as observed in *Arabidopsis* (i.e. 5 members)^17^. The SAG12 group is comprised of a single gene with two introns in *Arabidopsis*, whereas eight members with a single intron were found in both castor bean and physic nut. As shown in Fig. 2, the SAG12 group is obviously clustered into four subgroups: Subgroup 1 (Ia) includes *JcSAG12H1–2* and *AtSAG12*; Subgroup 2 (Ib) includes *JcSAG12H3–6* and *RcSAG12H1–3*; Subgroup 3 (Ic) includes *RcSAG12H4–5*; and Subgroup 4 (Id) includes *JcSAG12H7–8* and *RcSAG12H6–8*. Since members of all four subgroups can be found in other plant species (see below), castor bean and physic nut promise to have lost Ic or Ia subgroup members, respectively; and, the unique *AtSAG12* is more likely to be the result of massive gene loss after WGDs. As for other subfamilies, most members in castor bean and physic nut were shown to harbor one to three orthologs in *Arabidopsis*, however, the orthologs of *RcRD21B*/*JcRD21B*, *RcCEP2*/*JcCEP2* and *RcXBCP3L*/*JcXBCP3L* have also been lost in *Arabidopsis*. Thereby, it is probably safe to say that the ancestral Euphorbiaceae genome contained 20 PLCP family genes, i.e., three RD21s, two CEPs, two XCPs, two XBCP3s, one THI, five SAG12s, three RD19s, one ALP and one CTB.

### Evolution of the PLCP gene family in castor bean and physic nut

As discussed above, the PLCP family genes in castor bean and physic nut promise to evolve from 20 ancestors, and gene-specific expansion and/or loss was shown to be restricted to the SAG12 subfamily. Although the exon-intron pattern between orthologs is highly conserved, *RcXBCP3* has obtained an additional intron close to the 3′ untranslated region (Fig. 4). As expected, the sequence length and nucleotide substitution of introns are relatively more variable than that in exons between orthologs. Nevertheless, as shown in Fig. 2, the origin and evolution of subfamily members still needs to be resolved. The available genomes of several representative plants allow us to discuss this issue. When taking advantage of the castor bean, physic nut and *Arabidopsis* PLCP genes to trace their orthologs in these plants, we are able to find one RD21 and three CTBs in a single celled green alga, *Chlamydomonas reinhardtii* ^52^; one RD21, one RD19, one ALP and one CTB in spikemoss (*Selaginella moellendorffii*), an ancient vascular species first appeared at about 400 Mya^53^; one RD21, one CEP, two XCP, one XBCP, four SAG12s, one RD19, one RD19, one ALP and one CTB in *Amborella trichopoda*, a sister species to all other flowering plants^54^; two RD21s, one CEP, two XCPs, one XBCP3, four SAG12s, two RD19s, one RD19, one ALP and one CTB in rice (*Oryza sativa*), a model species of monocotyledons^55^; one RD21, one CEP, two XCPs, two XBCP3 (including one XBCP3L), one THI, four SAG12s, one RD19, one RD19, one ALP and one CTB in *Aquilegia coerulea*, a basal species of most eudicot clade^56^.

As shown in Fig. 2, the RD21 subfamily contains five OGs: Group I includes *RcRD21A*, *JcRD21A*, *AtRD21A* and *AtRD21B*; Group II includes *RcRD21B* and *JcRD21B*; Group III includes *RcRD21C*, *JcRD21C* and *AtRDL1*; Group IV includes *AtRD21C*, *AtRDL2* and *AtRDL3*; and, Group V includes *AtRDL4*, *AtRDL5* and *AtRDL6* (Supplementary Table S2). Group I members are widely distributed, which can be traced back to *C. reinhardtii* ^52^. The divergence of other groups is more likely to occur in the common ancestor of core eudicots, which was proven to experience the whole-genome triplication γ event^9^. Plant species not having undergone any recent WGD were found to contain one *RD21A* ortholog, one *RD21B* ortholog and one *RD21C* ortholog, e.g. papaya, cacao and grapevine^43–45^. And species such as poplar and cassava that have experienced one recent WGD^24,47^ contain one or two orthologs for *RD21A*, *RD21B* and *RD21C*, respectively. However, Group II is more likely to be lost in Brassicaceae, e.g. *A. thaliana*, *A. lyrata*, *Brassica rapa* and *B. oleracea* ^41,50,57,58^. In contrast, Groups IV and V were shown to be restricted to Brassicaceous plants probably resulted from the β event or fast evolution, though gene-specific expansion and/or loss were found in *B. rapa* and *B. oleracea* (Supplementary Table S2).

The CTB subfamily can also be traced back to *C. reinhardtii* ^52^, and a single member was found in most plant species, especially those not having experienced recent WGDs. In contrast, gene expansion was found in *C. reinhardtii*, poplar, cassava and Brassicaceous plants, resulted from recent WGDs and local duplication^24,38,45,50^. In *A. thaliana*, *AtCTB3* was produced from *AtCTB2 via* the α WGD, which is shared by *A. lyrata*, *B. rapa* and *B. oleracea*; *AtCTB1* was produced from *AtCTB2 via* tandem duplication, which is only shared by *A. lyrata* ^41,50,57,58^ (Supplementary Tables S1 and 2).

The CEP subfamily contains two OGs: Group I includes *RcCEP1*, *JcCEP1*, *AtCEP1*, *AtCEP2* and *AtCEP3*; and, Group II includes *RcCEP2* and *JcCEP2*. Group I members are relatively primitive, which can be traced back to *A. trichopoda* ^54^. This group was shown to be highly expanded through WGD and local duplication in Brassicaceous plants. By contrast, Group II is more likely to be derived from the γ event, and gene-specific loss occurred in Brassicaceous plants^41,50,57,58^ (Supplementary Table S2).

The XCP subfamily also includes two OGs: Group I includes *RcXCP1*, *JcXCP1* and *AtXCP1*; and, Group II includes *RcXCP2*, *JcXCP2* and *AtXCP2*. Like the CEP subfamily, Group I of the XCP subfamily can also be traced back to *A. trichopoda* ^54^, while Group II is more likely to be resulted from the γ event (Supplementary Tables S1 and 2).

The XBCP3 subfamily contains two OGs: Group I includes *RcXBCP3*, *JcXBCP3* and *AtXBCP3*, which can be traced back to the ancestral angiosperm; and, Group II includes *RcXBCP3L* and *JcXBCP3L*, which can be traced back to *A. coerulea*, though specific gene loss occurred in Brassicaceous plants (Supplementary Table S2). Interesting, Group II is highly expanded in poplar, resulted from the recent WGD and local duplication^24^ (Supplementary Table S2).

The THI subfamily usually contains a single member, which can be traced back to the ancestor of eudicots. However, specific gene loss was found in poplar^24^ (Supplementary Table S2).

The RD19 subfamily contains three OGs: Group I includes *RcRD19A*, *JcRD19A*, *AtRD19A* and *AtRD19B*; Group II includes *RcRD19B*, *JcRD19B* and *AtRD19C*; and, Group III includes *RcRD19C*, *JcRD19C* and *AtRD19D*. Groups I and III can be traced back to spikemoss^53^, whereas Group II is more likely to be resulted from the γ event (Supplementary Tables S1 and 2). In grapevine, Group II is highly expanded through local duplication^43^.

The SAG12 subfamily contains two main groups, i.e. SAG12 and PAP. The origin of the PAP group is still not clear, since it was only found in castor bean, cassava as well as Brassicaceous plants (Supplementary Table S2), which belong to two distinct plant families. In Brassicaceae, the PAP group is highly expanded *via* the α WGD, tandem duplication and transposed duplication^51^. The SAG12 group can be further divided into four orthologous subgroups: Ia can be traced back to *A. trichopoda*, and has been lost in castor bean, papaya, cacao and grapevine; Ib is more likely to appear in the common ancestor of core eudicots along with the γ event, and has been lost in Brassicaceous plants; Ic may also appear along with the γ event, and has been lost in physic nut, cacao as well as Brassicaceous plants; and, Id is more likely to be Euphorbiaceae-specific^14,21^ (Supplementary Table S2).

In addition to gene copies and exon-intron structures, expression divergence was also observed between orthologs/paralogs. The transcript level of several OGs such as *RcRD21A*/*JcRD21A*/*AtRD21A*, *RcRD19A*/*JcRD19A/AtRD19A*, *RcRD19B*/*JcRD19B/ AtRD19C*, *RcALP1*/*JcALP1*/*AtALP* and *RcCTB1*/*JcCTB1*/*AtCTB3* is highly abundant in leaf, flower and seed. In contrast, the paralogs of *AtRD21A*, *AtRD19A*, *AtALP* and *AtCTB3*, i.e., *AtRD21B*, *AtRD19B*, *AtALP2* and *AtCTB1*/*AtCTB2*, are considerably less expressed, though they are also constitutively expressed in these tissues^59^. As for two OGs of subfamilies CEP and XCP, which were generated along with the γ event, expression divergence is even more obvious. Among them, *RcCEP1*/*JcCEP1* and *RcXCP2*/*JcXCP2* have become the dominated isoforms in all tested tissues (Figs 5 and 6). Similar expression pattern can also be observed for their orthologs in *Arabidopsis*, e.g. *AtXCP1* and *AtXCP2* ^59^. It is worth noting that, *RcTHI1* represents the most abundant gene in the male flower of castor bean, which is in accord with the expression pattern of its ortholog in *Arabidopsis* (i.e. *AtTHI1*); by contrast, the expression level of its ortholog in physic nut (i.e. *JcTHI1*) is considerably low in flower bud (Figs 5 and 6).

## Conclusions

The first genome-wide analysis of PLCP family genes in castor bean and physic nut was performed in the present study, resulting in 26 or 23 members respectively. The phylogenetic analysis assigned them into nine subfamilies. Novel groups or subgroups, which are absent from *Arabidopsis*, were identified in RD21, CEP, XBCP3 and SAG12 subfamilies. Their orthologs are widely distributed in core eudicots, suggesting gene-specific loss occurred in *Arabidopsis* and other Brassicaceous plants. Moreover, the evolution characteristics of castor bean and physic nut PLCP family genes were also compared and discussed. Our findings provide a useful reference to characterize PLCP genes and analyze the family evolution in Euphorbiaceous plants and other species.

## Materials and Methods

### Identification and manual curation of PLCP genes in castor bean and physic nut

The genomic data for castor bean^6^ and physic nut^8^ were downloaded from Phytozome v11^60^ or NCBI (http://www.ncbi.nlm.nih.gov/), respectively. 31 *Arabidopsis* PLCPs obtained from TAIR10^61^ were used as queries for the homologous search. Sequences with an E-value of less than 1e^−5^ in the TBLASTN search^62^ were collected, and the positive genomic sequences were predicted using GeneMark.hmm^63^. The predicted gene models were further confirmed with transcriptome data available in NCBI, including cDNAs, ESTs and RNA sequencing reads. The presence of the Peptidase_C1 domain in deduced proteins was checked using SMART^64^. Gene expression annotation and definition of tandem/proximal duplications were performed as described before^14,46^. The alternative splicing (AS) isoforms were identified using Cufflinks (v2.2.1)^65^. The BRH (Best Reciprocal Hits) method^66^ was used to define orthologs across species, and the systematic name was assigned based on the best *Arabidopsis* ortholog.

### Multiple sequence alignment and phylogenetic analysis

Sequence alignment of full-length PLCPs was conducted using MUSCLE^67^. The unrooted phylogenetic tree based on the alignment was constructed using MEGA6^68^ with the maximum likelihood method, where the bootstrap was set to 1,000 replicates. Gene structures were displayed using GSDS (http://gsds.cbi.pku.edu.cn/).

### Analysis of sequence feature and conserved motifs

Protein properties were determined using the online tool ProtParam (http://web.expasy.org/protparam/) and subcellular localization was predicted using iPSORT (http://ipsort.hgc.jp/). Conserved motifs were analyzed using the MEME Suite (http://meme-suite.org/tools/meme) as described before^14^.

### Gene expression analysis

Global gene expression profiles over various tissues were examined based on Illumina RNA sequencing data available in NCBI SRA, i.e., root (SRX750579), leafage (SRX750580), mature leaf (SRX1097498), flower bud (SRX1037655) and seed (SRX750581) in physic nut; expanding leaf (ERX021378), male developing flower (ERX021379), developing endosperm II/III (ERX021375), developing endosperm V/VI (ERX021376), developing seed (SRX485027) and germinating seed (ERX021377) in castor bean. Read mapping was performed using Bowtie 2^69^, and the FPKM (fragments per kilobase of exon per million fragments mapped)^70^ was used to determine the gene expression level as described before^46^.

## Electronic supplementary material

Supplementary information
