# Supplementary material for: Genome-wide comparative analysis of papain-like cysteine protease family genes in castor bean and physic nut
Source: Sci Rep. 2018 Jan 10;8:331. doi: 10.1038/s41598-017-18760-6 (PMC5762910; doi:10.1038/s41598-017-18760-6)
Supplement: Supplementary file 1 — Supplementary information [file 41598_2017_18760_MOESM1_ESM.pdf]

Genome-wide comparative analysis of papain-like cysteine protease family genes in castor bean and physic nut

Zhi Zou[1*], Qixing Huang[2], Guishui Xie[1] & Lifu Yang[1]

[1] Key Laboratory of Biology and Genetic Resources of Rubber Tree, Ministry of Agriculture, Rubber Research Institute, Chinese Academy of Tropical Agricultural Sciences, Baodaoxincun, Danzhou 571737, Hainan Province, China

[2] Institute of Tropical Biosciences and Biotechnology, Chinese Academy of Tropical Agricultural Sciences, Xueyuan Road 4, Haikou 570100, Hainan Province, China

[*]Correspondence and requests for materials should be addressed to Z.Z. (Email: zouzhi2008@126.com or zouzhi@catas.cn)

Supplementary Files:

Supplementary File S1. The gene model for *RcRD21C*. The coding region is marked with uppercase letters, above which are its deduced amino acids. The transcribed untranslated regions, including 5' UTR, intron and 3' UTR sequences, are marked with lowercase letters. The start and stop codons are marked with bold letters.

Supplementary File S2. The gene model for *RcXCP1*. The coding region is marked with uppercase letters, above which are its deduced amino acids. The transcribed untranslated regions, including 5' UTR, intron and 3' UTR sequences, are marked with lowercase letters. The start and stop codons are marked with bold letters.

Supplementary File S3. The gene model for *RcXBCP3*. The coding region is marked with uppercase letters, above which are its deduced amino acids. The transcribed untranslated regions, including 5' UTR, intron and 3' UTR sequences, are marked with lowercase letters. The start and stop codons are marked with bold letters.

Supplementary File S4. The gene model for *RcXBCP3L*. The coding region is marked with uppercase letters, above which are its deduced amino acids. The transcribed untranslated regions, including 5' UTR, intron and 3' UTR sequences, are marked with lowercase letters. The start and stop codons are marked with bold letters.

Supplementary File S5. The gene model for *RcPAP3*. The coding region is marked with uppercase letters, above which are its deduced amino acids. The transcribed untranslated regions, including 5' UTR, intron and 3' UTR sequences, are marked with lowercase letters. The start and stop codons are marked with bold letters.

Supplementary File S6. The gene model for *RcCTB1*. The coding region is marked with uppercase letters, above which are its deduced amino acids. The transcribed untranslated regions, including 5' UTR, intron and 3' UTR sequences, are marked with lowercase letters. The start and stop codons are marked with bold letters.

Supplementary File S7. The gene model for *JcSAG12H8*. The coding region is marked with uppercase letters, above which are its deduced amino acids. The transcribed untranslated

regions, including 5’ UTR, intron and 3’ UTR sequences, are marked with lowercase letters. The start and stop codons are marked with bold letters.

Supplementary File S8. The gene model for *JcRD21B*. The coding region is marked with uppercase letters, above which are its deduced amino acids. The transcribed untranslated regions, including 5’ UTR, intron and 3’ UTR sequences, are marked with lowercase letters. The start and stop codons are marked with bold letters.

Supplementary File S9. The gene model for *JcCEP2*. The coding region is marked with uppercase letters, above which are its deduced amino acids. The transcribed untranslated regions, including 5’ UTR, intron and 3’ UTR sequences, are marked with lowercase letters. The start and stop codons are marked with bold letters.

Supplementary File S10. The gene model for *JcXBCP3L*. The coding region is marked with uppercase letters, above which are its deduced amino acids. The transcribed untranslated regions, including 5’ UTR, intron and 3’ UTR sequences, are marked with lowercase letters. The start and stop codons are marked with bold letters.

Supplementary File S11. The gene model for *JcTHI1*. The coding region is marked with uppercase letters, above which are its deduced amino acids. The transcribed untranslated regions, including 5’ UTR, intron and 3’ UTR sequences, are marked with lowercase letters. The start and stop codons are marked with bold letters.

Supplementary File S12. The gene model for *JcSAG12H1*. The coding region is marked with uppercase letters, above which are its deduced amino acids. The transcribed untranslated regions, including 5’ UTR, intron and 3’ UTR sequences, are marked with lowercase letters. The start and stop codons are marked with bold letters.

Supplementary File S13. The gene model for *JcSAG12H2*. The coding region is marked with uppercase letters, above which are its deduced amino acids. The transcribed untranslated regions, including 5’ UTR, intron and 3’ UTR sequences, are marked with lowercase letters. The start and stop codons are marked with bold letters.

Supplementary File S14 The gene model for *JcSAG12H7*. The coding region is marked with uppercase letters, above which are its deduced amino acids. The intron sequences are marked with lowercase letters. The start and stop codons are marked with bold letters.

Supplementary Figures:

Supplementary Fig. S1. Detailed information of 15 motifs identified in this study.

Supplementary Fig. S2. Matched positions of 25 RcPLCP genes on physic nut chromosomes. The positions were based on the synteny analysis and only 8 matched chromosomes were shown. RcPLCP genes were shown behind that of physic nut.

Supplementary Tables:

Supplementary Table S1. Detailed information of 31 PLCP genes in *Arabidopsis*. [1] "Yes" represents genes containing alternative splicing isoforms; [2] "S, M and C" represent signal peptide, mitochondrial targeting peptide or chloroplast transit peptide, respectively, which was predicted by iPSORT; [3] duplicated modes were determined based on the study of Wang et al. (2013).

Supplementary Table S2. List of orthologous groups of the PCLP gene family based on the analysis of representative plant species. "OG" represents "orthologous group". "-" represents "Not Detected".

Supplementary File S1. The gene model for *RcRD21C*. The coding region is marked with uppercase letters, above which are its deduced amino acids. The transcribed untranslated regions, including 5’ UTR, intron and 3’ UTR sequences, are marked with lowercase letters. The start and stop codons are marked with bold letters.

1 tccatttatcctcttatactcctatttcttcgagttaggtcactaaattaattaacattt

1 M

61 cttgcaaaatttgcattcctcttataaatatacacataccccaatctcccttactcttc**A**

2 Y N P L L K N Q N S F H N I S T M A L P

121 **TG**TATAACCCTCTTCTCAAAAACCAAAATTCTTTCCACAACATATCAACCATGGCATTAC

22 I S L S T L L F L F F T L S S A W D M S

181 CCATATCATTATCAACCCTTCTCTTCCTTTTCTTCACTCTATCATCTGCCTGGGACATGT

42 I L S H N H G H H H Q S S W R S D N E V

241 CCATCCTCAGCCATAACCATGGCCATCATCATCAATCAAGCTGGAGAAGTGATAATGAGG

62 I S M Y N W W L A K H S K T Y N K L G E

301 TCATTAGCATGTACAATTGGTGGCTTGCAAAGCATAGTAAAACGTACAATAAGCTAGGTG

82 R E K R F E I F K N N L R F I D E H N N

361 AGAGAGAGAAGAGGTTTGAGATCTTCAAGAATAACTTGAGGTTTATTGATGAACACAACA

102 S K N R T Y K V G L T R F A D L T N E E

421 ACTCTAAGAATAGAACATACAAGGTTGGATTGACTAGGTTTGCTGATCTTACTAACGAGG

122 Y R A K F L G T K S D P K R R L M K S K

481 AGTACCGTGCCAAATTCTTGGGCACAAAGAGTGATCCTAAGCGTAGATTAATGAAGTCCA

142 N P S Q R Y A F K A G D V L P E S I D W

541 AAAACCCAAGTCAGAGATACGCTTTTAAGGCCGGTGATGTGTTGCCGGAATCCATTGACT

162 R Q S G A V S A I K D Q G S C G

601 GGAGGCAGAGTGGAGCTGTTAGTGCGATCAAAGATCAAGGAAGCTGCGgtaagcaatttc

661 tatatcaattactaattagaaaactacatgaaaaatgctacatacagttattagatataa

721 aaaataatagaagattaatcaaggtatctcaatgcaatttttttttttttgagttgcttgg

781 aagaagaatggtggttaattttctggtcatgcagaaaggtagtgaagaatggtagttact

841 ggatcttctagttagctagtggattcttttcagattacttataatatatatgtagggatt

901 gtcccataaatgtcacaaaaatataaaataattataaaaatattcttgtacatttctgat

961 aataaaaatactaaaaacagaattttatttataaaaatactctaaaatcgagtcgagttt

1021 tatttataaaaatatccgatttgatttttttcaaattaaagggtatatgttggatatattt

1081 tgaaaaaaagtatatatattcagtataattttggtaaataataaatttaaaatatattaa

1141 tttttataaaatattattgtagtttgtttgattttgtaatattaataatgaataaataaa

1201 taaataaatttaaaagataatttttaatttaaaaatttgaagatgcattgagaatattat

1261 atccaaaaaactagataaattgctataagataaaaaaaaatgtttagtatataatgaaga

1321 aaaaaagtnnnataaaataaatataaatttaagaaaatcgaacgataaaaatatatacat

1381 ttgctaaataatagtatatttgaagtatttgaaactttttaaaataaaataaatttcaaa

1441 ctcaaaatgttctaacatcaaaccagtgtatgtttcttaaaaccatggtatacgttagat

1501 atgtgtaaaatattttaaactaattttgaaaaaataaaaaagataaaaaaagtaaagaat

1561 tggtaattatgaaaagtgaagtacttttataattatttaaaaaaagataaataaagaaat

1621 ttcctctaatatttaaggtttgagtttgagcccaggatccagagcccattaagtcaaaac

1681 acccagatctatatctaaacttctaagtttcagtttgagtccagacccaagttaaagtca

1741 acttggactaaggctagtgtcaacttaatgagcgagatagcgaccccaccaattgggtgg

1801 ccacagttctggattcacggttccattttcattggaaccggatcgaaagaccaattccaa

1861 gccggtttcagtttacagttatggttcgatcagtttagatatatttaagaaaataaaaaa

1921 agagtttaaactcaattatacaattattaaatacttattaaatattaattacataattat

1981 taagtattaattacatagcattatattaattaataatttaattgttgtctttttttaaatt

2041 ttaaggggttggaacctaaatggatagttctgattccatctaaaggaaaatcggacttga

2101 accgacctccacagtactgagctgattcaagccaggtttcaaaattgaccaatttcaagc

2161 tggttttaaccagctccaatttcgaaccggttcgtggccaccactacacatcaatgttga

2221 gcgagattcaaaattaacttagttcaatctttaggccaatctaattatcaaaattaaaaa

2281 aattattaggaatactcatgtttttttatcaaatttatggtttttttttttccatttttatt

2341 gtgttaaaaacaattgaaaataattgagaaacaactaataccaacagaaaaaagattacc

2401 acactctaaatttttttaaaaatttaaaactgtacttaaaaaatatttatacaataaaaat

2461 gatatttttttgttaattctaatatatttttgaaagattctctataagatttttcggttta

2521 gatcagcttagttttatcctttttttcttgaaataatttgacttaattttgttatcaaatc

2581 caactcatatattggaagtacacacaaaaattatattgaattgcttatgaacttttttt

2641 tttttttttttgtgttcttatcaaataatttgaattgtttatgaactattctttgtgtt

2701 tctatcaaaaaaaaaattagactgaacttcaaaattgatacattttagatgatctgatag

2761 gtctagtgtgggggcacctaatggagttgcataactcttgctataacaaaaagacttatc

2821 aaatttttttttttaaaaaaaaagttaaaacattattatggaagagaatcagtaaatcaa

178

2881 attcaggtactttcagcacaagaataaaataaatatgcaatgccattgtgttggtgcagG

178 S C W A F S T I A A V E G V N K I V T G

2941 TAGTTGCTGGGCATTCTCAACAATAGCTGCAGTAGAAGGCGTAAACAAGATAGTAACAGG

198 E L I S L S E Q E L V D C D R S Y N A G

3001 GGAGCTAATATCTTTATCAGAACAAGAACTTGTAGACTGCGACAGGTCATACAATGCAGG

218 C N G G L M D N A F Q F I I N N G G I D

3061 TTGCAATGGAGGTCTGATGGATAATGCTTTCCAGTTCATCATCAACAATGGTGGCATTGA

238 T D K D Y P Y Q A V D G K C D T T K

3121 CACCGATAAAGACTATCCATATCAAGCTGTTGACGGCAAATGTGACACTACCAAGgtatt

3181 tgaagtgtatattactaaggtcattgatcacttctaatcttttcattgtttgttttgggc

256 V K N K A V T I D G F E

3241 gtttattattttggattgtttcagGTGAAGAATAAGGCCGTCACCATTGATGGGTTTGAG

268 D V M A F D E M A L Q K A V A H Q P V S

3301 GATGTTATGGCTTTCGATGAGATGGCCTTGCAGAAAGCTGTGGCACATCAGCCTGTCAGT

288 V A I E A S G M A L Q F Y Q S

3361 GTTGCCATTGAAGCCAGTGGCATGGCTTTACAGTTCTACCAGTCGgtgagttctttccca

3421 tttctttcaattcttgcagagaaatgagtttcttgttccttatctattttcctgaaacat

3481 tagttatcaaagtaattgcagaaagtttttaaattctctttttgagagataacattggt

3541 gagacatggaagtggtgtcattttgcttgtatgttaaaacaccaattgagtagaaacgtt

303 G V F T G E C G S A L

3601 gagttagttttcttttaatttttcagGGTGTATTCACTGGTGAATGCGGATCAGCCCTA

314 D H G V V I V G Y G T E D G I D Y W L V

3661 GACCATGGAGTGGTTATTGTTGGCTATGGAACAGAAGATGGTATAGATTATTGGCTTGTT

334 R N S W G R D W G E N G Y I K M Q R N V

3721 AGGAATTCATGGGGCCGTGATTGGGGTGAAAATGGATATATCAAAATGCAGCGTAATGTG

354 V D T F T G K C G I A M E S S Y P I K N

3781 GTTGACACATTTACTGGCAAATGTGGAATTGCGATGGAGTCTTCGTATCCAATCAAGAAT

374 T Q N P V K I S S V *

3841 ACCCAAAATCCTGTTAAGATCAGCAGTGTT**TGA**atttgtccttttggttctctgtaaagc

3901 aaagggcaccaagagaaggaaaaaacaagaagatcaaaggtgcaaaatcttatccttctt

3961 cttagtctaaagggcaagaaggcgttatgcgattgtatgaatgattgttgttgtatataa

4021 gctttgaatgtgtcaaaacagcaccttaaaaatgagctgatcgtggaatctggattgcat

4081 tttctctgaagttgattgttctaataaaacttaccgagcacataaatatgcagtcgttat

4141 cttttctgcatttgttaattgcttgctagctgaattgcttaatagaccaaaggtgcctag

4201 gcagtagggccaggctcagtagacaccaagaggagagagttagtggagacagggcacaat

4261 ttcttctctgaaaccaaacaaatcaacaagaagaaagaaacccaaaagagcgagcaaaga

4321 tctccatcaaaatgcatgatttcttctccaactttctacatgtgcaataaagttaaacaa

4381 acttgctatctgtaagcttttagaattctttttatattgataattgccaactttttcag

4441 tccaaaaacaatatcccatgacttctttttaggcattggctatgttaagttagaaacgca

4501 cacaaaaaaaaaacaatgaagcattaagtttgcttgatagataaggtgcctactatcaat

4561 tccatcatcagactggaagcggctcttacaaggggcaacttttgtttttctgttattat

4621 tatttttcttttaattgttgccgcgcttagagatggatttt

Supplementary File S2. The gene model for *RcXCP1*. The coding region is marked with uppercase letters, above which are its deduced amino acids. The transcribed untranslated regions, including 5’ UTR, intron and 3’ UTR sequences, are marked with lowercase letters. The start and stop codons are marked with bold letters.

```
  1 tctattattactttatcaatatgcttatgtccctaaagcagaccaacaatcttggggcaa
 61 aagcgtaccaaaaagaaccaaccgtaaagattcggtatgcattgaaaattaatttcagct
121 acttcactttaaagcaatttaagaaattaaaacctgtatataactgctgcccggctccct
  1                                                  M  A  P
181 ccatcttcattcatctaaccctcccttaccattaagcctcaacctaaataaATGGCTCCC
  4 S  V  S  S  I  F  L  F  T  I  F  T  S  L  V  I  C  S  V  V
241 TCAGTTTCAAGCATTTTCCTTTTCACCATTTTCACATCTCTCGTCATCTGTTCAGTTGTT
 24 A  H  D  F  S  I  V  G  Y  S  P  E  H  L  T  S  M  H  K  L
301 GCTCATGACTTTTCTATTGTGGGTTACTCGCCGGAGCACTTGACTTCCATGCATAAACTC
 44 T  E  L  F  E  S  W  M  S  K  H  G  K  T  Y  E  S  I  E  E
361 ACCGAGCTCTTCGAATCATGGATGTCTAAGCACGGCAAAACCTACGAAAGCATTGAAGAG
 64 K  L  H  R  L  E  V  F  K  D  N  L  M  H  I  D  R  R  N  R
421 AAGCTACACAGACTCGAGGTTTTCAAAGATAACTTGATGCACATCGATCGTAGAAACAGG
 84 D  V  T  T  Y  W  L  A  L  N  E  F  A  D  L  S  H  E  E  F
481 GATGTTACGACCTACTGGCTTGCGCTGAATGAGTTTGCAGACTTGAGCCATGAAGAGTTC
104 K  S  K  Y  L  G  L  N  T  D  A  P  R  G  R  I  S  E  D  L
541 AAGAGCAAGTACTTAGGGTTGAACACCGATGCTCCCAGGGGGAGAATTTCTGAAGACTTA
124 S  Y  R  D  V  I  D  L  P  K  S  V  D  W  R  K  K  G  A  V
601 AGTTACAGAGATGTAATAGACTTGCCCAAATCCGTCGACTGGAGAAAGAAAGGAGCTGTT
144 A  P  V  K  N  Q  G  S  C  G  S  C  W  A  F  S  T  V  A  A
661 GCTCCAGTCAAGAACCAAGGCTCGTGCGGCAGCTGTTGGGCATTCTCAACAGTTGCAGCG
164 V  E  G  I  N  Q  I  V  T  G  N  L  T  S  L  S  E  Q  E  L
721 GTTGAGGGCATAAACCAGATTGTGACCGGAAATTTGACTTCACTGTCCGAGCAGGAGTTG
184 I  D  C  D  T  S  F  N  S  G  C  N  G  G  L  M  D  Y  A  F
781 ATCGACTGCGACACGTCCTTTAACAGTGGCTGCAATGGAGGCCTCATGGATTATGCATTT
```

204 D Y I V N N G G L H K E E D Y P Y L M E

841 GACTATATCGTCAACAATGGAGGACTCCACAAGGAGGAAGACTACCCATACCTAATGGAA

224 E G T C D E K R

901 GAAGGCACTTGTGATGAAAAGCGGgtaaaagggcattgcaaaaaccattgttttacagta

961 taggctaattctaaactggactcaattgtcaaatgactagtaaaattgtgctaacatttc

232 E E M E V V T I S G Y H D V

1021 tatggctattgtcttcagGAAGAGATGGAGGTAGTGACTATCAGTGGTTACCATGATGTG

246 P E N N E E S L L K A L A H Q P L S I A

1081 CCAGAAAACAACGAAGAAAGCCTCTTGAAGGCACTAGCTCACCAACCTCTTAGCATAGCT

266 I E A S G R D F Q F Y G R

1141 ATTGAGGCTTCTGGGAGAGATTTCCAGTTCTACGGCAGGgtaagaaaattgacatgcaca

1201 cacacacacatctgataaatttcgtagcgggtaagaccacatttcagaaaaaatttctaa

279 G V F N G P C G T D L D H G V

1261 acagagggatctggcagGGGGTATTCAATGGACCTTGTGGGACAGATCTAGATCATGGAG

294 A A V G Y G S S K G L D Y I I V K N S W

1321 TAGCCGCAGTCGGATATGGATCGTCAAAGGGGCTAGATTACATCATCGTGAAGAATTCCT

314 G P K W G E K G Y I R M K R N T G K P E

1381 GGGGACCAAAGTGGGGTGAAAAAGGCTACATACGAATGAAGAGAAACACAGGAAAACCAG

334 G L C G I N K M A S Y P T K K K *

1441 AAGGCCTATGTGGGATCAACAAAATGGCTTCATATCCTACTAAAAAGAAG**TAA**cacagca

1501 gggttgacaacatagtatttcctttctttctgatagctccttcattgatcaaactttctt

1561 aatccttgttggtactcttatcaataaaatcagatagaaaattcacatccctcaaatcct

1621 tgtgctattaaaaagtagataatggcattacacatgtaaacaagaatgcagacaagagga

1681 tgtgcatgcttatgtgcacaaacaatcatgtttctaacagagagtcaaataaaataaaga

1741 tgcagatgagcatgaatagcaaaatgaagttctatatatcagggtttatttaacttcttt

1801 attaattcaatgatcataaattagtagtaaatacatgcaagtttaaggtgacaaggacct

1861 aaaacatgtgtatattgatgattggctgctaatttaatggtttaacgatagagcagaaca

1921 agtatcttaactcaaaacctcattgtacttgaaaacacagtcataaagccggcctccaag

1981 aaactgggcaacctctggatgtttcacc

Supplementary File S3. The gene model for *RcXBCP3*. The coding region is marked with uppercase letters, above which are its deduced amino acids. The transcribed untranslated regions, including 5' UTR, intron and 3' UTR sequences, are marked with lowercase letters. The start and stop codons are marked with bold letters.

```
  1 ccacgtttttaggcagccaatccaataaaaagaaaatacacgcccatactaaaaaggcaa
  1                                                M  N  F  L  S  A
 61 gattccctttatttcctccgagagaaaaataagaaaaaagaaaaATGAACTTTCTTTCTG
  7  L  F  L  I  T  L  L  F  F  N  L  S  I  S  S  F  S  S  S  S
121 CTCTTTTCTTGATCACTCTCCTCTTCTTTAACCTTTCTATTTCTTCCTTTTCTTCTTCTT
 27  D  I  S  K  L  F  E  S  W  T  K  E  H  G  K  T  Y  T  S  K
181 CTGATATCTCTAAGCTCTTTGAGAGTTGGACCAAAGAACATGGAAAAACATACACTTCAA
 47  E  D  K  L  Y  R  F  K  I  F  E  E  N  Y  E  F  V  K  K  H
241 AGGAAGATAAGTTGTATAGATTCAAAATCTTCGAGGAAAATTATGAATTTGTAAAGAAAC
 67  N  S  Q  G  N  S  S  Y  T  L  S  L  N  A  F  A  D  L  T  H
301 ATAACAGCCAAGGTAATTCTTCTTATACCCTTTCTCTCAATGCCTTTGCTGATCTCACCC
 87  H  E  F  K  A  S  R  L  G  L  S  A  F  S  T  S  G  K  L  S
361 ACCATGAATTCAAAGCTTCTCGCTTGGGCCTCTCTGCTTTTTCAACCTCCGGGAAGCTTT
107  R  R  N  F  P  L  H  D  F  V  G  D  V  P  I  S  I  D  W  R
421 CCCGTCGGAATTTTCCATTGCATGATTTCGTTGGCGATGTTCCTATATCAATTGATTGGA
127  K  K  G  A  V  S  Q  V  K  D  Q  G  N  C  G
481 GAAAGAAAGGGGCTGTTTCTCAGGTCAAAGATCAAGGGAATTGCGgtgcgtgcatctttt
541 atttggattctttatctttttatgcttaattatttgatgagtttttgaactactctagt
601 ctttctttatattgatccatgagcatcgtgcatacttagttttgaacttcttgtgtgatc
661 tttattatcatttattagtagtaatctcttaactctgtttttccttctacttattaaact
721 tgggaatcatgtaagaattgcagccatcttatttattagatttcgtaatatagtctagta
781 cctcagcatgctgtataccggcatttgattttgattgttttgtcaatgaaatatacatct
841 ctctatatttcttaatcgtattccaaaaagggatttgccacctttataattttaaaatcc
901 tcatcaaagagatggtggcttgtgaaaaatgctttcatttatagatttgttttaccttga
961 aagttttctcctttttagttttgattgcagtgaagttcagttcttcacaatttttttctca
```

1021 tgcactatatttttcttctgatattcttagttaagacttccatcttgactgaaatcatca

1081 agttagtactatttcttgttgcgttcttagttatcaagactttcactttgactgaaatca

1141 tcaagttctacaattgactgtaatctggttaaatcattctactatgactaaatgatatat

1201 atatggccataattgttagctgtttttgactggtaaattgcagatacatctatttcatgg

142 A C W S F S A T

1261 tgcttaagaataaattgtttactgtattacgcagGTGCTTGTTGGTCGTTCTCAGCCACA

150 G A I E G I N K I V T G S L V S L S E Q

1321 GGAGCTATTGAAGGCATAAATAAGATTGTTACTGGATCTCTCGTTAGCCTCTCGGAGCAG

170 E L V D C D R S Y N N G C E G G L M D Y

1381 GAGTTAGTTGATTGCGACAGATCTTACAATAATGGTTGTGAGGGTGGACTAATGGACTAT

190 A Y Q F V I E N N G I D T E E D Y P Y Q

1441 GCCTACCAGTTTGTCATAGAAAACAATGGGATTGACACTGAGGAAGATTATCCATATCAA

210 A R E K T C N K E K

1501 GCACGGGAAAAGACCTGCAACAAGGAAAAGgtaattattttatttccatttgctttcctt

1561 cacctataaactctcaattcagttcccactcttccgtgttcacatcattgtctatgttga

220 L K

1621 ttcactctctgtttctcgtttcattccggtagtcctaatttatacacattgcagTTGAAA

222 R H V V T I D G Y T D V P Q N N E K E L

1681 AGGCATGTGGTAACCATTGATGGATACACTGATGTGCCCCAAAACAATGAGAAAGAGTTA

242 L K A V A A Q P V S V G I C G S E R A F

1741 TTGAAAGCGGTTGCAGCTCAACCTGTAAGTGTCGGTATATGCGGCAGTGAGAGAGCATTT

262 Q L Y S K

1801 CAGTTGTACTCAAAGgttagtttttgatagttaatatcaatgaccatttactatagacag

267 G I

1861 caaaaaaaaaattggttagtggatttttaagtggtttaattatttgtatgatagGGAATT

269 F T G P C S T S L D H A V L I V G Y G S

1921 TTCACTGGTCCATGTTCAACTTCTTTGGATCATGCTGTATTGATTGTAGGATATGGGTCA

289 E N G V D Y W I V K N S W G T H W G I N

1981 GAAAATGGTGTAGATTATTGGATTGTGAAGAACTCATGGGGAACACACTGGGGAATAAAT

309 G Y M Y M L R N S G N S Q G L C G I N M

2041 GGTTATATGTACATGCTACGCAACAGCGGCAATTCTCAAGGGCTCTGTGGTATCAACATG

329 L A S F P V K T S P N P P P P A P P G P

2101 CTGGCTTCATTTCCAGTGAAGACCAGCCCTAATCCCCCTCCTCCAGCTCCACCAGGACCC

349 T K C D L F T R C G E G E T C C C T R R

2161 ACTAAATGTGATCTTTTCACTCGATGCGGAGAAGGGGAAACTTGCTGTTGTACTCGCCGC

369 I F G L C F S W K C C E L D S A V C C K

2221 ATTTTTGGATTATGCTTTTCGTGGAAGTGTTGCGAATTGGATTCAGCTGTGTGTTGCAAG

389 D G L H C C P H D Y P V C D T K R N M C

2281 GACGGGCTCCACTGTTGCCCCCATGACTATCCAGTTTGCGACACAAAAAGGAACATGTGC

409 L K

2341 CTTAAGgtcagcatattctctgccttcaatctgctagctgtttgattgtgtgatgcacat

2401 aaaactatcaaagctaggcatgattatactgtcgtttgaatttcttctgaacattaaatt

2461 gcttccttgacattcttccttttatttttgtaacttcaaagatcttactctctggtggaa

2521 atgatagaattgcatgggttgtctttttgtttatcccacgtgatgttcaatactgtggat

2581 aaaaccacaaatttgatagagttgatttatcattttagctaaaatctatctgatgttgtg

2641 attctataaagtgcaattaaagcttgaaattaagtttgtcttcagtagactgatactgct

411 F P G N A T R M E T V A K K S S S

2701 tatgcttgcagTTTCCTGGTAATGCCACAAGAATGGAAACAGTTGCAAAGAAAAGCTCCT

428 G M F G S W N S L L E G

2761 CTGGGATGTTTGGCAGTTGGAATTCCCTTCTTGAAGgttggattctgtaaaaccattttc

2821 atttattctggcgagtgatttaatttttcagaaaaaacccttgtagaagttgcatgttt

2881 cacaattgaagggagggagactttctagatttagagcgtaaattttgtgtacaccccaga

2941 agttgtattgttgtagcttgtgtgtagagagatttctggtgcatggcattgttttacaat

3001 ggggggatggatttaagaagggaattaaggaggtgagcttggcattgtttcttctttcta

3061 gagtccatgaatttaagaattgattagggattttccttatcctagatataagactgcttt

3121 tagcatatgtctttcgtatgtctgtacgtatattacgtctcccgagctgcttacctgcat

3181 ctttatttgtgtgcatcaattgcttgaatattagatataaagatatcctatcagctagtt

440 T A I A K G

3241 ttgatatgtaagcgagtagataagcaaacctctatgaacagGTACAGCTATTGCAAAAGG

446 H P R I A G V I V C Q D N I I S F R H Q

3301 CCACCCTAGGATTGCTGGAGTAATTGTGTGCCAAGACAATATCATATCCTTCAGGCACCA

466 N *

3361 AAAT**TAA**aacacacgcaaaaaaaaatgaaaaagaacaccagaaaagaggtgagaacatca

3421 ttctgcctggataattaggggttgctgcagggaccttgatgatttctctctagacctgtc

3481 atgggcatggaaaaacaagccctgtgcacctagacagaactcttttaacaactggcagtg

3541 tgaatgtgaatttaccataagagtcccaattggaacttgttgacttcttccagtatcgaa

3601 ctcttcacccaaatttatcaggcactctgtccagttctgccattgatgatgagtctctgc

3661 cattgatgatcgtcgtttatcaaatttgtaaactgccaaagtacgtaaatgagaatatga

3721 tgctgttccagcctgagacataattcaatcattcacataactcttcattaaaa

Supplementary File S4. The gene model for *RcXBCP3L*. The coding region is marked with uppercase letters, above which are its deduced amino acids. The transcribed untranslated regions, including 5’ UTR, intron and 3’ UTR sequences, are marked with lowercase letters. The start and stop codons are marked with bold letters.

1 M G S Q K I

1 ggtagatcaaagaaatcatcaccggaaacccaatttgaggaaa**ATG**GGTTCCCAAAAGAT

7 Q F L I I F L L V G P L T C L S F T L P

61 CCAATTCCTGATCATTTTCTTGCTTGTAGGTCCACTAACATGCCTATCTTTTACCCTCCC

27 D E Y S I V G N D L H E L L S E E R V K

121 AGATGAATACTCTATAGTGGGCAATGACCTTCATGAGCTGCTATCTGAGGAACGTGTCAA

47 E L F Q Q W K E K H R K V Y K H V E E A

181 AGAACTCTTTCAGCAATGGAAAGAAAAGCACAGAAAGGTTTACAAGCATGTAGAGGAAGC

67 E K R L E N F R R N L K Y V V E K N Q K

241 AGAAAAGAGGCTAGAAAACTTTCGAAGGAACTTGAAGTATGTAGTGGAGAAGAATCAGAA

87 K K N L G S A H T V G L N K F A D M S N

301 GAAGAAAAACTTGGGCTCAGCGCATACAGTTGGATTGAATAAGTTTGCTGATATGAGCAA

107 V E F R Q K Y L S K V K K P I K K R N N

361 CGTGGAGTTTAGGCAAAAGTATCTTTCAAAAGTGAAAAAACCCATCAAGAAACGGAACAA

127 N L M T S R Q R N L Q S C V A P S S L D

421 TAATTTAATGACCAGCAGGCAGAGAAATTTACAATCATGCGTTGCTCCTTCAAGCTTGGA

147 W R K K G V V T P V K D Q G D C G

481 TTGGAGAAAGAAAGGAGTTGTTACTCCAGTTAAGGATCAAGGAGATTGTGgtatgtttct

541 ttaatttctttcttttgttattttctttataattatattatctgttttattttcagttgc

601 tgttgtattggacttagtggatactaacattaattaattggaaaaaagaaattcaaatac

661 ttttgaatgcaacgaagatcataataaattaagtaatagattcttgaatgtcatgttgac

721 agataatcttaatttttaatggaatatccaaagatttagtcgtttttattagtaaaagtt

781 ttatttaaattgcaatgctgctttataagtaatttcatataattattatgagaaagagag

841 tatctaatgcttaaatttaaggcttgtttagatgaataaagatggtttataatttcagat

901 ctaaaggaaagaagagtctggtttgactggtaggtacgcactagggttgaatcagtgtcg

961 tattagattcttgttgctggggtgttgtataaaagtaatttgagtgaaagtaaataataa
1021 taataattaatcggatggtgtatctgtaagactttttaactgtaaatttaagaatattat
1081 ttttagaaatttcgttttgctctttataattaagtgattattacactgtttttaatttaa
1141 aaaacatttataaagtgaaagtaaacaaaagaacagacatttaattgcaaatttaagcat
1201 tttagttttagtaattttatttagactttatcattaagtgattattactgtttttagtt
1261 taaaaaacatttataagcgaaagataataatactgctcaattggctatatgcttccatcc
1321 acctgcatatttttgtgtggtcaaaaaggaaaacatttccaaaaaaaaggtccaactaga
1381 tatagagaaattctaaagaaaaagcagatatgtttctcattgcacctaaagtatatatta
1441 ataagtatgagtttgtttggtataaataaattatttaaaactttttttgataagttatgt
1501 agtttatgatttgataaattatttttagataaaataaaatacattaaaaaaattttacta
1561 ttaaaaaatatcaatttctagagatttaattatttttactttaatcaactattattatca
1621 tattaaatatatttgctttaacaattcaaaaatattaccgtgtgattgtcaagttggtct
1681 cgtcagaccaatgacttcttaaatcacaatttgacaattaattaataaaattatgaatta
1741 gtttctaaacttattaaacatgtgaaaacataataataataataataataataataacct
1801 gattaaagtgaaaatagtttcacttcttgctatccaaagtttttttaacagtaaaacacat
1861 ttactgctatacattataaccacatttttctttttaactaagaaaaaatacattaaaaat
1921 cataaaacagaacaagtacagtttaatactttatctacattattaattttatgaagttaa
1981 aaattacagtttaaatcttaaagtgacaagaaaatttgtatgtggaaagtaaacgttacc
2041 ttttgcatcacctatgttattgcttttatcatgagtatacagtttaatactttatctaca
2101 ttaggtgaattattaattttatgagcctctttatatatctcgtaattattgggatttcat
2161 gaactaaaaaaataaaaagtaatagaaatatctatttgctttatttttttaatcaaattta
2221 caatatttttttatttttattatgttaaggtattaaaaaaatatgatttttttaaaaattag
2281 aattttttatttcttttgttgtttttgagcaaaataattaaaaaataaccgaaaaataat
2341 tatactataaatttgnnnnnnnnnnnnnnnnnnnnnnnnnnnnnnnnnnnnnnnnnnnnnn
2401 nnnnnnnnnnnnnnnnnnnnnnnnnnnnnnnnnnnnnnnnnnnnnnnnnnnnnnnnnnnn
2461 nnnnnnnnnnnnnnnnnnnnnnnnnnnnnnnnnnnnnnnnnnnnnnnnnnnnnnnnnnnn
2521 nnnnnnnnnnnnnnnnnnnnnnnnnnnnnnnnnnnnnnnnnnnnnnnnnnnnnnnnnnnn
2581 nnnnnnnnnnnnnnnnnnnnattatactataaatttgaaaaggagacagtattttaaaag

2641 aaaattatacagtaaaaacaatattctttttattgattttaaggtatttaaatatttttg

2701 aaaaaattcctaaaaaacatacagcttaaatttttgaaatcaccggattgatcctggcag

2761 ctcaacgaacaagtctaataacacttcagcatcctaactaaattattgaaacttgcaagt

2821 accaaaatactaaattagtggtgctcctgcttcattttaactaaaactgatcaatattcg

2881 gatatcttcactcgtccacaagcttattaacagtttcatttatttttaatgttttgtttt

164 S C W A F S

2941 aggtgttttgtaattgaggtcttggttggttaattttgcagGAAGCTGTTGGGCATTCTC

170 S T G A I E G I N A I V T G D L V S L S

3001 TTCCACTGGAGCAATCGAAGGAATAAATGCCATTGTCACCGGAGACCTCGTTAGCCTTTC

190 E Q E L M D C D T T N Y G C D G G Y M D

3061 TGAACAAGAACTTATGGATTGTGATACAACCAATTACGGTTGTGATGGTGGCTATATGGA

210 Y A F E W V I N N G G I D T E I D Y P Y

3121 CTACGCTTTTGAATGGGTTATAAACAATGGTGGGATTGATACAGAAATCGATTATCCTTA

230 T G V D G T C N I A K

3181 TACAGGTGTGGATGGTACCTGCAACATCGCCAAGgttcgtttcccttcgtgttctatata

3241 acccactagaagatgattataatatcttttcttcgtcttacttgtggtgttcttgtgttt

241 E E T K V V S V D G Y E D V A E S D

3301 gatcagGAAGAAACAAAAGTTGTATCCGTTGATGGATACGAAGATGTAGCTGAATCAGAT

259 S A L L C A T V Q Q P I S V G I D G S A

3361 AGTGCTCTCTTGTGTGCTACTGTTCAGCAGCCTATTAGTGTCGGTATCGATGGTTCTGCA

279 I D F Q L Y T S

3421 ATAGATTTTCAACTGTACACAAGTgtaagtccctctttcattttatgagtagcacccaaa

3481 aaaaaaaaaaaaaaaatcaaaagatacttaaagaagtgattatgacatgaaagatgatgtg

287 G I Y N G S C S D N P N D I D H A

3541 atttaatcagGGAATCTACAATGGTAGTTGCTCTGATAATCCAAATGACATTGATCATGC

304 V L I V G F G S E D G E D Y W I V K N S

3601 TGTTTTGATCGTTGGATTTGGCTCTGAAGATGGTGAAGATTATTGGATCGTGAAGAATTC

324 W G T E W G I N G Y F Y L L R D T N L P

3661 ATGGGGCACAGAATGGGGAATAAACGGATATTTCTATTTATTAAGAGACACTAATTTGCC

344 Y G V C A V N A M A S Y P T K E S S A P

3721 ATATGGTGTTTGTGCAGTCAATGCTATGGCCTCTTATCCCACTAAAGAATCCTCTGCACC

364 S P T S P P S P P S P P P P P P P T P V

3781 ATCCCCGACTAGTCCTCCATCACCACCATCACCCCCTCCACCTCCACCCCCAACACCAGT

384 P P P P S P S P S E C G D F S Y C P T D

3841 GCCCCCACCTCCCTCACCTTCACCAAGTGAATGTGGCGACTTCTCTTATTGTCCAACTGA

404 E T C C C L Y E F F D F C L V Y G C C P

3901 CGAGACATGTTGTTGCCTTTATGAATTCTTTGATTTCTGCTTAGTCTACGGTTGCTGTCC

424 Y E N A V C C T G T E Y C C P S D Y P I

3961 GTACGAGAATGCTGTTTGCTGCACTGGCACAGAATATTGTTGCCCCAGTGATTACCCAAT

444 C D I K E G L C L Q

4021 TTGTGATATTAAAGAAGGGCTCTGTCTTCAGgtgagaactcatagccattaatatctgag

4081 aattgcctctttctagcgtgaataacgagctattagttcaaaatacttgcaagttacgaa

4141 ttattaagccattattcaatcagttgattgaatggttaacctttctttttccaaaattgt

454 N Q G D Y L G V A A T K K H M A K

4201 gtatgcaacagAACCAAGGAGATTACTTGGGAGTAGCTGCAACGAAGAAGCATATGGCTA

471 H K L P W S K L E E S K R E R T Y Q P L

4261 AACACAAGCTTCCCTGGAGCAAGCTAGAAGAAAGCAAGCGAGAGAGAACATACCAACCTC

491 M W K R N P F A A I R *

4321 TCATGTGGAAGAGGAACCCTTTTGCTGCAATTCGC**TGA**aagaagaaaagctgatggcccg

4381 ttaagtaattgaaggtcttcgcaattattttaattaaatttaccgcataggcagacattc

4441 agtaatcctgctacaattattggtttatgaacattaaacgaatgtagcaagaaaaactag

4501 aagtaaagaagaaaaggggaaattgagtatggcgttctcttggatgatcgactggatgga

4561 aactggatttgttagtaagacttctttcatgtattttctcatggcttacagctcatttca

4621 agccatgctctgtattgttgtccaggacttgtaatatacttcagtatatggttttaaaat

4681 aagagattctgtttggaagtttacttgatttcaatttcagaaaactgatctttac

Supplementary File S5. The gene model for *RcPAP3*. The coding region is marked with uppercase letters, above which are its deduced amino acids. The transcribed untranslated regions, including 5' UTR, intron and 3' UTR sequences, are marked with lowercase letters. The start and stop codons are marked with bold letters.

1 M A L S L E K K L A I A L L V V F S T W

1 **ATG**GCTCTATCACTTGAAAAGAAACTCGCGATTGCCTTGTTGGTGGTTTTCTCGACATGG

21 A S Q A M A R Q L I N E D A L V E K H E

61 GCATCTCAAGCCATGGCCCGACAGTTGATCAATGAAGATGCCCTCGTTGAGAAGCACGAG

41 Q W M A R H G R T Y Q D S E E K E R R F

121 CAATGGATGGCTCGCCATGGGCGCACTTATCAGGACAGTGAAGAAAAAGAGAGACGGTTT

61 Q I F K S N L E Y I D N F N K A S N Q T

181 CAGATATTCAAGAGCAACTTAGAGTACATTGACAACTTCAACAAGGCCTCCAATCAGACT

81 Y Q L G L N N F A D L S H E E Y V A T Y

241 TACCAGTTAGGTCTCAATAATTTTGCAGACTTATCCCATGAAGAATACGTTGCTACTTAC

101 T A R K M P V L P T A N T T T K T T L F

301 ACTGCACGCAAGATGCCCGTACTCCCTACAGCAAACACCACAACCAAGACAACGCTATTC

121 N S D D I N Q V E V P E S I D W R D H G

361 AATAGTGATGATATTAACCAGGTTGAGGTTCCGGAGAGTATTGACTGGAGAGACCATGGC

141 A V T P I K N Q Y Q C G

421 GCTGTCACCCCTATTAAGAACCAATACCAATGTGgtaacatctcatctctctttacgtta

481 tatatatatataaaattaagtgggaattattaatgaattcaacgggtttatttagttgag

541 aagtgaatttgcttatacaaaatagtaaaatatatgtaaaactttctaaaagaatcactg

601 acttggagatgtcaatctagtatttgaaatgggtaccaactaaataaagagtttctgtct

153 C C W A F

661 ttctttgactgattaccctccttatatattgtgtattgcacgcagGGTGTTGCTGGGCAT

158 S A A A A V E G I V A N G V S L S A Q Q

721 TTTCAGCGGCGGCAGCCGTGGAAGGGATCGTAGCTAATGGTGTCTCACTGTCAGCTCAAC

178 L L D C V S D N Q G C K G G W M N N A F

781 AATTACTAGACTGTGTGAGTGATAATCAAGGTTGTAAGGGGGGTTGGATGAATAATGCTT

198 N Y I I Q N Q G I A L E T D Y P Y Q Q M

841 TTAATTACATAATACAAAACCAGGGCATAGCCTTAGAAACGGATTACCCGTATCAACAAA

218 Q Q M C S S R M A A A Q I S G F E D V T

901 TGCAACAAATGTGCAGTTCAAGAATGGCTGCAGCCCAGATAAGTGGTTTTGAAGATGTAA

238 P K D E E A L M R A V A K Q P V S V T I

961 CTCCAAAAGACGAGGAAGCCCTGATGAGGGCAGTAGCAAAGCAACCTGTATCGGTCACCA

258 D A T S N P N F K L Y K E G V F T A A G

1021 TTGACGCTACTTCTAATCCAAACTTCAAATTGTACAAAGAGGGAGTGTTCACTGCAGCAG

278 C G N G H S H A V T L V G Y G T S E D G

1081 GCTGCGGGAATGGACATAGCCATGCTGTGACCTTAGTTGGGTATGGGACAAGTGAAGATG

298 T K Y W L A K N S W G E T W G E S G Y M

1141 GGACCAAGTATTGGCTAGCCAAGAATTCATGGGGTGAGACTTGGGGAGAAAGTGGTTACA

318 R L Q R D I G L E G G P C G I A L Y A S

1201 TGAGGTTGCAGAGAGATATTGGTTTAGAAGGAGGTCCTTGTGGCATTGCATTGTATGCTT

338 Y P T I N *

1261 CGTATCCTACCATTAAT**TAG**acccgctctatctaagaatattacaacatgcataaaataa

1321 aagaaaagac

Supplementary File S6. The gene model for *RcCTB1*. The coding region is marked with uppercase letters, above which are its deduced amino acids. The transcribed untranslated regions, including 5' UTR, intron and 3' UTR sequences, are marked with lowercase letters. The start and stop codons are marked with bold letters.

```
  1 cttgctttgtactatctatctgtatctaccaataaattaatcaaagcctcattaaccgaa
 61 cacactttccttattcgacgtttactatatagctccttcctgccttgttgatactgcggc
  1                     M  A  A  S  I  L  S  S  F  A  L  L  L
121 agttgctttggagaagcaaaaATGGCAGCTTCTATCTTATCCTCCTTCGCTCTTTTGCTG
 14 F  L  V  A  L  S  S  F  H  S  R
181 TTTCTTGTGGCTCTCTCCTCTTTTCACTCCCGGgtacctattattgttgttattagtatt
241 tcttttgtctggtttataaagtttctgatcgtgctgattaaatttagttttggatttgaa
301 attaagaatgacgagattgttttaactcatcgatttcgatttctgtagcttgaatttaag
361 agttgatgaagttatttcaacggattgatttcgattttgtacctatgtcatctgtgccga
421 aagtaaaatttcatgtaaccgaagttttatttgaactaaaaatgtaaaggaacgtaccta
481 tttatgctactgtgatgatcacctctctaataagcactgtaaagccacatgaatttatct
541 tatataggagtgtttagagtttgtggatttatgttgcgccttttgttgctgttgttgtct
601 tgtgcttgctcctttgcataccctgataagaggaggatgttcattcttgaatgttcttca
661 aatcttgttctgagttttgattcttttggaggttactgtttgactttttaaacatcctaa
 25                      V  I  S  T  E  L  D  S  K  L  K  L  N
721 atgctgttgacactgtcaacagGTGATCTCAACAGAACTAGATTCCAAGCTGAAGCTGAA
 38  S  R  I  L  Q
781 CTCTAGGATCCTTCAGgtattttttctttgctgccgatcattggaatccaaaagctttatt
 43                                                 E  S  I  I  K
841 ctgtagagcaagtcatttaattgctttttctgacttcgacgagcagGAGTCAATAATTAA
 48  K  V  N  E  N  P  D  A  G  W  E  A  A  M  N  P  Q  L  S  N
901 GAAAGTAAATGAAAACCCTGATGCTGGATGGGAAGCTGCCATGAATCCTCAACTTTCCAA
 68  F  T
961 CTTCACTgtgagctttaactcatcagaggccaacatctttttctaagtccatttgtctac
 70                                        V  G  Q  F  K  Y  L  L  G
```

1021 agttaacacatacttctatattttgctgcgtagGTTGGCCAATTCAAGTATCTTCTTGGA

79 A K P T P K K E L M G V P M I S H P K T

1081 GCCAAGCCAACTCCCAAAAAGGAACTGATGGGTGTTCCTATGATAAGTCATCCAAAAACC

99 L K L P K E F D A R T A W P H C S T I G

1141 TTAAAGTTGCCAAAAGAATTTGATGCAAGAACAGCTTGGCCACATTGCAGTACCATTGGA

119 K I L D

1201 AAAATTCTAGgtcagctgctttctttctataatatattttcaattttcttcttcctttttt

1261 cttgaggtgctgatgtttccactatgtatgccaaatgcttgattctggatcaatttcctc

123 Q

1321 atcaaagATCAGgtaaaatctggttctattgttttctcagtcaactcactcaacatgagt

124 G H C G S C W A

1381 tctacatattctgagttcgtctgtatatgacaatccagGGTCATTGTGGATCCTGTTGGG

132 F G A V E S L S D R F C I H F G M

1441 CATTTGGTGCTGTTGAATCACTATCTGATCGATTTTGTATCCATTTTGGCATGgtaacta

1501 gctagctatattcactaaatctcctgttaaacttcactagctgtgttgtccattacaaac

149 N I S L S V N D L L A C C

1561 atatcttttgttttgccacagAATATATCATTGTCTGTCAATGATCTCTTGGCATGCTGT

162 G F L C G D G C D G G Y P M Y A W R Y F

1621 GGCTTTTTGTGTGGAGATGGTTGTGATGGAGGGTATCCGATGTATGCATGGAGATACTTT

182 V H H G V V T E E

1681 GTCCACCACGGGGTTGTCACTGAGGAGgtacaaatttctgtgtttgcctgtttccttggt

1741 tcttctgttttcccctgcactggagattgttaaattggaagctaagggttcttgtaatgt

1801 catgtttctttttttgatgacttcatgaatataattatttgatgcaatggcttcttctctt

191 C D P Y

1861 gtttttaaatatttcttctaaaaatctgcaaactgtttacatctgacagTGTGACCCATA

195 F D N I G C S H P G C E P G F P T P K C

1921 TTTTGATAACATTGGCTGTTCCCACCCTGGTTGTGAGCCTGGATTTCCAACTCCAAAGTG

215 V R K C I D K N Q L W R Q S K H Y S V N

1981 TGTGAGAAAGTGCATTGATAAGAATCAGCTCTGGAGGCAGTCAAAGCATTACAGCGTTAA

235 A Y R I S S D P H D V M A E V Y K N G P

2041 TGCATATAGAATTAGTTCTGATCCCCATGATGTTATGGCTGAAGTTTATAAGAATGGACC

255 V E V S F T V Y E

2101 AGTAGAGGTTTCTTTCACTGTTTATGAGgtaacatatcaattcgtgtcttcttgtttgct

2161 tgcattaacttctttgtatgtgaatataagcttatttcttttgctgttctatccctgctg

2221 taatccttaaccataattaagtgagctgaagaaatcttgagccagcttcacatgtagatg

2281 taggaagaacctcaatgggagttttcatatattggatgcctgaaatatgattatcttttc

2341 catgtatattagatactcgaacttttcatgataatagctcttggtagaattactttttg

264 D F A H Y K S G V Y K H

2401 aagctactgctttgcgttctacagGATTTTGCTCATTATAAATCTGGAGTTTATAAGCAC

276 I T G E V M G G H A V K L I G W G T S D

2461 ATTACAGGTGAAGTAATGGGAGGTCATGCAGTTAAGCTTATTGGGTGGGGGACTTCTGAT

296 N G E D Y W

2521 AATGGAGAGGATTATTGGgtatgcgtgattagctaactttttttgcagatattttagctat

302 L L A N Q

2581 gaccttataaggatgttggtataattgtgcacttttctttcttgcagCTTCTTGCAAACC

307 W N R G W G D

2641 AGTGGAATAGAGGCTGGGGAGATgtatgttcctataataagtttcttgctgatgaatcac

2701 gctactttcccttcttttgtttttctcttcatccttttattcccctgctctcaaattttc

314 D G Y F K I R R G T N E

2761 acaaatttttatttcgaatgacagGATGGTTACTTTAAGATAAGGAGAGGAACAAATGAG

326 C G I E D D A V A G L P S A R N L D L V

2821 TGTGGTATTGAAGATGATGCGGTTGCTGGTTTGCCTTCAGCCAGAAATCTTGATCTTGTT

346 R E V A S M D A L E D A F A *

2881 AGAGAGGTTGCCAGCATGGATGCTCTTGAAGATGCTTTTGCT**TGA**tagtagatcattgta

2941 cttgcaagttctgtaatttacgcatcagtcatgagttacagagatatgttatgggtgtgt

3001 ctgctgttcaggcatgaaacgaaaccatatttatgaatccatgctgtgttggtgcttata

3061 tcaactagacatttgttgtgaatgcaagaaaggaacaaatctgtaaataatgtaattgtc

3121 acacatagtataagagtcaactgctttctgctttcacattgcttttacattaggcactta

3181 tgaaatgtttggaaggatgtccaaattaataagatagtcctgctttatgaagaaaatggg

3241 aagtttctgcagaaatagctctaattgtcagtagcataagccctaacccgagagatggaa

3301 tctaatcgtggtacgcccaacagaaggagtacatgtggtcggctagcaagcgagtcaaat

3361 actgtttaaggataaggtagtgactcttccttttgctttttccccggaggtgggggcta

3421 tataggaaaacattgtaggtaataatatttttgtttttgcacttacagccagcatgttca

3481 ttgtaggcttagtattccgttttataatgttatattttcccctcatcattccaa

Supplementary File S7. The gene model for *JcSAG12H8*. The coding region is marked with uppercase letters, above which are its deduced amino acids. The transcribed untranslated regions, including 5' UTR, intron and 3' UTR sequences, are marked with lowercase letters. The start and stop codons are marked with bold letters.

1 gaaactgaattttggttgtccatatcatacacaactaacgggattaactcaacataagac

61 tgcaatttcagacatctcttgtaaggaagccaatttatatgttctataaatacatgacag

121 ctctttaaggactagtgtgccaaaaatacacatatcctacattatatactctctagtctt

1 M A S I L E N K L V F V T

181 tacttcctcctccaccaagaaga**ATG**GCTTCAATTCTTGAAAACAAACTGGTCTTTGTGA

14 V L V L G L F A S Q A F A R P L Q N E T

241 CAGTGTTGGTGCTGGGGCTCTTTGCATCTCAAGCCTTTGCACGTCCTCTACAAAATGAAA

34 M K E R H E M W M A K Y G R V Y K D S A

301 CCATGAAGGAGAGGCATGAAATGTGGATGGCTAAATATGGACGTGTTTATAAAGACAGTG

54 E K E K R F N I F K N N V E F I E S F N

361 CAGAGAAGGAGAAACGCTTTAACATATTCAAGAATAATGTGGAGTTCATTGAATCTTTTA

74 K D G N K L Y K L D I N G F A D L S N E

421 ATAAGGATGGGAATAAGCTTTACAAGCTAGATATCAATGGATTTGCAGACTTAAGTAATG

94 E F K A S R N G Y K R S S I A K S S E T

481 AAGAGTTTAAGGCCTCTAGAAATGGATATAAAAGATCTTCTATTGCTAAGTCATCTGAGA

114 L S F K Y E N V T A V P T S M Y W R N K

541 CGCTGTCGTTTAAGTATGAAAATGTCACCGCTGTCCCAACTTCCATGTACTGGAGGAACA

134 G A V T P I K D Q G Q C G

601 AAGGAGCTGTTACTCCCATCAAGGACCAAGGCCAATGCGgtaaggaacacaatgtttctt

661 atttttcttctactatgtactagctatagtaaaattaatctaatatatgtatttcccttt

147 C C W A F S A V A A M E G I

721 tggaacacacaaatgtagGATGTTGCTGGGCATTTTCTGCTGTGGCTGCCATGGAAGGAA

161 T K L S T G K L I S L S E Q E L V D C D

781 TTACAAAGCTCTCAACAGGAAAAATTGATTTCTCTTTCTGAGCAAGAACTTGTTGATTGTG

181 T S G E D Q G C E G G L M D D A F E F I

841 ACACAAGTGGAGAAGATCAAGGCTGCGAAGGGGGTCTTATGGATGATGCATTTGAATTCA

201 K K N G G L T T E A N Y P Y Q G T D D S

901 TAAAAAAAAATGGAGGACTAACAACTGAAGCCAATTACCCTTACCAAGGAACTGATGATT

221 C N K R K A V D H A A K L T G Y E D V P
961 CTTGCAATAAAAGAAAGGCAGTTGATCACGCAGCAAAGCTTACTGGCTATGAAGATGTGC
241 A N S E D A L L K A V A N Q P V S V A I
1021 CTGCTAATAGTGAAGATGCCTTATTGAAGGCAGTAGCCAACCAACCAGTCTCTGTAGCCA
261 D A S G S A F Q F Y S G G V F T G D C G
1081 TTGATGCAAGTGGCTCTGCTTTCCAGTTCTACTCAGGTGGAGTATTTACAGGAGATTGCG
281 T E L D H G V T A V G Y G T S S D G T K
1141 GAACTGAACTCGACCATGGAGTTACTGCAGTTGGGTATGGCACAAGCAGTGATGGAACCA
301 Y W L V K N S W G T S W G E N G Y I R M
1201 AGTATTGGTTAGTGAAGAACTCATGGGGAACTTCTTGGGGTGAAAATGGATACATTAGAA
321 E R D I D A S E G L C G I A M E P S Y P
1261 TGGAAAGAGATATTGATGCTAGCGAAGGTCTTTGTGGAATTGCGATGGAACCTTCTTACC
341 T A *
1321 CAACTGCA**TAA**ttaaaattaagtggcagaaaagaaaatatgggtcaatcttaaaatataa
1381 atacattgtccaaaa

Supplementary File S8. The gene model for *JcRD21B*. The coding region is marked with uppercase letters, above which are its deduced amino acids. The transcribed untranslated regions, including 5’ UTR, intron and 3’ UTR sequences, are marked with lowercase letters. The start and stop codons are marked with bold letters.

```
  1 ctttaccggtaacctgtctttgtacatgtagtaatccgattctaaaaatataaatttctc

 61 cctatatatatacgctcaaaagcacaaagcaaaatcacccatatcaccatccgacccaac

  1           M  A  S  P  G  R  S  L  P  F  F  I  L  C  G  F  L

121 cctacagccATGGCTTCTCCTGGAAGATCTCTCCCTTTTTTCATCCTATGTGGGTTCCTT

 18 F  F  S  S  L  A  L  D  M  S  I  I  D  Y  N  I  R  H  G  Q

181 TTTTTCTCCTCTTTAGCGCTGGACATGTCGATTATTGATTACAATATCAGGCATGGACAA

 38 N  P  E  E  R  T  D  A  N  V  R  R  I  Y  E  M  W  L  V  K

241 AACCCAGAAGAAAGAACAGACGCTAATGTTCGGAGAATCTATGAGATGTGGCTCGTCAAA

 58 H  G  K  A  Y  N  A  L  G  E  K  E  K  R  F  Q  I  F  K  D

301 CATGGGAAAGCTTATAATGCTTTGGGCGAGAAGGAGAAGCGGTTTCAGATCTTTAAAGAT

 78 N  L  K  F  I  D  E  H  N  S  V  N  R  S  Y  K  L  G  L  N

361 AATCTTAAATTCATCGATGAGCATAACTCGGTTAATCGGAGTTATAAGCTCGGATTGAAC

 98 R  F  A  D  L  S  N  E  E  Y  R  A  M  F  L  G  T  R  M  E

421 CGGTTCGCCGATCTGAGTAATGAGGAGTACCGGGCGATGTTCTTGGGTACAAGGATGGAA

118 R  K  N  R  L  G  A  S  M  G  T  E  S  R  Y  L  Y  K  E  G

481 AGGAAAAATAGATTGGGGGCATCGATGGGGACTGAAAGTAGGTATTTGTATAAAGAAGGT

138 D  D  L  P  E  K  V  D  W  R  E  K  G  A  V  V  P  V  K  N

541 GATGATTTGCCGGAAAAGGTTGATTGGAGAGAAAAAGGGGCTGTTGTTCCTGTCAAGAAT

158 Q  G  Q  C  G

601 CAAGGACAGTGTGgtaagtttgtttcttagcgatttttcctttttctttttttgagttttg

661 ttggttttgattgcttgtgaagtgggacctggaagcatggcatgttcttccaaggatttt

721 cctggaaactagcgacaccaattgatttttgatttgttagtgttatctccttagcaggga

163                                                        S  C

781 tatttcttgatttttcgtgaaagaaaataaattgtttgtttttatttattgaagGGAGTT

165   W  A  F  S  T  V  A  A  V  E  G  I  N  Q  I  V  T  G  D  L
```

841 GCTGGGCTTTCTCAACTGTTGCTGCAGTAGAAGGGATTAACCAGATTGTAACTGGTGATC

185 I S L S E Q E L V D C D R S Y N Q G C N

901 TAATCTCTCTATCAGAGCAGGAGTTGGTAGATTGTGATAGATCATATAACCAGGGATGCA

205 G G L M D Y A F E F I I Q N G G I D T E

961 ATGGAGGTCTTATGGACTATGCCTTTGAATTTATTATCCAAAATGGTGGTATTGATACTG

225 Q D Y P Y E A V D N V C D P Y R

1021 AACAAGATTACCCATATGAGGCTGTTGATAATGTTTGTGATCCATACAGGgtatgctaaa

1081 tttatttatttacttaatctttgttctgtagttacattggtatattgatcgttctaattt

241 K N A K V V T I D G Y E

1141 tatttgttgttcttgcatcttttagAAAAATGCTAAGGTAGTTACTATTGATGGATATGA

253 D V P E N D E K S L K K A V A N Q P V S

1201 AGATGTTCCTGAAAATGATGAGAAATCCTTGAAGAAGGCTGTGGCAAATCAGCCAGTTAG

273 V A I E A G G R A F Q L Y Q S

1261 TGTTGCCATTGAAGCTGGTGGCAGGGCTTTCCAACTTTACCAATCGgtaattactcttta

1321 ttgccaaatgaaattatctcatttgcagtttaacaagagcagtgttatattttgtgagta

1381 ctagttatacagaaactattataattcctattcttttggtttggcataattttgttaact

1441 caactcaatatatgttatgcatcagaatcatataacaagttgaatatataaagtacaaat

288 G V

1501 ctttgctggcattgattgttttgtacttgtctttaattttacttcctcaatgcagGGTGT

290 F T G R C G T Q L D H G V A A V G Y G T

1561 TTTTACTGGTAGATGTGGTACACAGCTTGACCATGGTGTGGCTGCTGTTGGATATGGCAC

310 E N G V D Y W L V R N S W G P D W G E S

1621 AGAGAATGGTGTAGATTACTGGTTGGTGAGGAACTCATGGGGTCCTGATTGGGGTGAAAG

330 G Y I K L E R N L A T T T T G K C G I A

1681 TGGTTATATCAAGTTGGAGCGTAATTTGGCCACCACCACAACTGGAAAATGTGGAATTGC

350 V E A S Y P T K K G P N P P N P G P T P

1741 AGTGGAGGCTTCATATCCAACCAAGAAGGGTCCAAATCCCCCTAATCCTGGCCCAACCCC

370 P S P V N P P P P K P S T Q C D D Y F S

1801 TCCATCTCCGGTCAACCCTCCTCCTCCAAAGCCCTCCACTCAGTGTGACGATTACTTTTC

390 C Q E G S T C C C I Y E Y G S Y C F G W

1861 ATGCCAAGAGGGAAGCACCTGCTGCTGCATATATGAGTATGGTAGCTATTGCTTTGGATG

410 G C C P L E S A T C C D D H Y S C C P H

1921 GGGATGCTGCCCTCTTGAGTCTGCAACCTGCTGTGATGACCACTATAGCTGCTGCCCTCA

430 D F P V C D L E A G T C R L

1981 TGATTTCCCTGTCTGCGACCTTGAAGCTGGAACCTGCCGATTGgtaaacaaaacatataa

2041 atcttatttttgtcaaagaaatttaaaagatgtttgataagtgtattgactttttttcttc

444 S K D N P L S V K P F R R S P

2101 ttttgctgatggcagAGTAAGGATAATCCATTGAGTGTAAAACCATTCAGACGTAGTCCT

459 A Q R I G S H L R G G K K I I G A *

2161 GCTCAAAGAATCGGTTCTCACCTTCGCGGTGGCAAGAAAATTATTGGTGCT**TGA**aagctc

2221 agggtctgcaggacattcagcaagtgaggcttaaacagagaattgtttcctattgcatca

2281 taggaggatttagcaaggaagaaatgctttttatctcaccctatttaaacaattactgaa

2341 tgtttgattttctttttttttttttttttcctttttatgatttattggtgactattgtaaa

2401 tactttcagtttgagctaaagcagaaactgaactataaccaattaggatatatagttcac

2461 ctgaatttgtaaagtcaccaactattgattctcattaattaagattgctggtttattaca

2521 tgcttgtgttaagatattttatatatgaagtaacatgattttcactggctggaatggaca

2581 cttga

Supplementary File S9. The gene model for *JcCEP2*. The coding region is marked with uppercase letters, above which are its deduced amino acids. The transcribed untranslated regions, including 5’ UTR, intron and 3’ UTR sequences, are marked with lowercase letters. The start and stop codons are marked with bold letters.

```
  1 ctttgattgtctgaagtcacgttttctttctgttgcatagccgtaacctttttctgctca
 61 atttttggttaaagctctcaaaaaggctataaccctcatctcttctccatggctatatat
121 acaattcaacacatccttgcaatttcattgctaagccaacaaagaaaatcaagaattccc
  1                                              M  G  A  R  N  I
181 gactttggtaacccttttcttgatttatagtaggtttagatattATGGGTGCAAGAAATA
  7  I  L  F  A  V  S  L  V  L  V  F  K  I  A  E  S  L  D  Y  Q
241 TAATCTTGTTTGCCGTTTCATTAGTGCTAGTTTTTAAGATAGCTGAAAGTTTGGATTACC
 27  E  A  D  L  A  S  E  E  S  L  W  N  L  Y  E  R  W  R  S  H
301 AAGAAGCAGACTTAGCGTCGGAAGAGAGTCTTTGGAACTTGTACGAGCGGTGGAGAAGCC
 47  H  T  I  S  R  S  L  T  E  K  K  Q  R  Y  N  V  F  K  E  N
361 ATCACACAATCTCTCGTAGCCTGACAGAGAAAAAGCAGCGATACAATGTGTTCAAGGAAA
 67  L  K  H  I  H  N  V  N  Q  K  D  K  P  Y  K  L  R  L  N  K
421 ATTTGAAGCATATTCATAACGTTAACCAGAAGGATAAGCCTTATAAATTGAGATTGAATA
 87  F  A  D  M  T  N  H  E  F  L  Q  H  Y  G  G  S  K  V  S  H
481 AGTTTGCGGATATGACTAATCATGAGTTCTTGCAACATTATGGAGGCTCTAAGGTTAGCC
107  F  R  M  L  H  G  S  R  Q  K  T  G  F  S  H  H  N  T  D  L
541 ACTTCAGGATGCTACATGGGTCTCGTCAGAAAACTGGGTTTAGCCACCACAACACTGATC
127  P  P  S  V  D  W  R  K  K  G  A  V  T  G  V  K  D  Q  G  K
601 TTCCACCTTCCGTTGATTGGAGAAAGAAAGGAGCAGTCACTGGTGTAAAGGATCAAGGCA
147  C  G
661 AATGTGgtgagtttctcaagtcataaatttcagctttcagtatttttagtaataattttg
721 aacttataatcactagatctttaaatgacactcttaactcttgtgtttataaatattaaa
149                            S  C  W  A  F  S  S  V  A  A  V  E
781 atcaatgatttttatgctatacagGTAGCTGTTGGGCATTTTCAAGTGTGGCAGCAGTGG
161  G  I  N  K  I  K  T  G  E  L  V  S  L  S  E  Q  E  L  V  D
```

841 AGGGTATTAACAAAATCAAAACAGGAGAATTAGTTTCTCTATCAGAGCAAGAGTTGGTTG

181 C S S A N H G C D G G L M E Q A F K F I

901 ATTGCAGCTCTGCCAACCATGGCTGTGATGGAGGATTAATGGAACAGGCATTCAAGTTCA

201 K K I G G I T A E T T Y P Y T A R D G S

961 TTAAGAAAATTGGTGGAATAACCGCAGAGACTACCTATCCTTATACTGCTAGAGATGGAT

221 C D S N K

1021 CCTGTGACTCAAACAAGgttaactgagtttaatattgatctataactcaactttttcagt

226 M

1081 gtacgtatatgtttctatgcacaagtgaagggattatatatctttcttggtgttgtagAT

227 N A P V V Q I D G Y E M V P E N D E N A

1141 GAATGCTCCTGTTGTGCAAATTGATGGATACGAAATGGTGCCAGAAAACGACGAGAATGC

247 L M K A V A N Q P V S I A I D A G G K D

1201 ATTGATGAAAGCTGTTGCTAACCAACCAGTATCTATTGCAATAGATGCAGGCGGAAAAGA

267 M Q F Y Y E

1261 TATGCAATTCTATTATGAGgcaagtttttttattcaataggaaactgtacagctgaatca

1321 aacagaccctatgatatagtttttttcattccctgaaaattttgtttctcttgttgttgca

273 G V F T G D C G T E L N H G V A L V G Y

1381 gGGAGTATTCACTGGAGATTGCGGGACAGAGCTAAACCATGGGGTAGCACTTGTGGGATA

293 G T T L D G T K Y W I V K N S W G S D W

1441 TGGCACAACACTAGATGGCACAAAGTATTGGATAGTGAAGAATTCATGGGGAAGTGATTG

313 G E N G Y I R M E R G I D A E E G L C G

1501 GGGAGAGAATGGTTACATAAGAATGGAAAGAGGGATTGATGCAGAAGAGGGACTGTGTGG

333 I T L E A S Y P I K N S S D N K K S P D

1561 TATAACTTTGGAGGCCTCTTACCCTATCAAGAATAGCTCAGACAACAAGAAATCACCCGA

353 S R R D E L *

1621 TTCTCGCAGAGATGAACTC**TAA**atttgattatatctatctatatatatatatgcctcaat

1681 aacttaaggacctaaaaggagagtttttaaatttaattaccgctttctcttttagtatta

1741 attaagttgaagttggatttctttgccatctcttttgttatatatagttcaatgatttga

1801 attccaaataaaaccaatgctttctctc

Supplementary File S10. The gene model for *JcXBCP3L*. The coding region is marked with uppercase letters, above which are its deduced amino acids. The transcribed untranslated regions, including 5’ UTR, intron and 3’ UTR sequences, are marked with lowercase letters. The start and stop codons are marked with bold letters.

```
  1 gaggatatgttgctctgactggtaggccgggagtaagaccatgtttcgaaacttataaac
  1       M  T  D  Q  N  P  I  G  H  C  L  V  I  K  R  K  M  S
 61 actataATGACAGATCAAAACCCAATCGGCCACTGCTTAGTGATAAAGAGAAAAATGAGT
 19 F  Q  K  L  G  L  A  I  V  F  L  V  L  S  L  V  P  L  T  C
121 TTCCAAAAGTTGGGATTAGCAATCGTTTTTCTGGTTTTATCTTTAGTGCCACTAACCTGC
 39 F  S  F  S  L  P  S  E  Y  S  I  V  G  N  D  L  H  E  L  L
181 TTCTCTTTTAGCCTCCCAAGCGAGTACTCTATAGTTGGAAACGACCTTCATGAGCTGCTT
 59 S  H  E  R  V  I  E  L  F  Q  Q  W  K  L  E  H  K  K  I  Y
241 TCTCACGAACGGGTGATCGAGCTGTTTCAACAATGGAAACTGGAGCACAAAAAGATTTAC
 79 K  H  A  E  E  A  E  K  R  L  E  N  F  R  R  N  L  K  Y  V
301 AAGCACGCAGAAGAGGCAGAAAAGAGGCTCGAAAATTTTCGAAGGAATTTAAAGTATGTG
 99 I  E  K  N  A  K  N  A  E  M  G  T  G  F  K  V  G  L  N  K
361 ATAGAGAAGAATGCGAAGAATGCAGAGATGGGTACAGGATTTAAAGTTGGATTGAACAAA
119 F  A  D  L  S  N  E  E  F  K  Q  T  Y  L  S  K  V  K  K  P
421 TTTGCAGATTTGAGTAATGAGGAGTTTAAGCAAACGTATCTGTCGAAAGTGAAAAAACCT
139 I  S  K  K  K  N  T  M  M  T  T  G  Q  R  N  L  Q  A  C  E
481 ATCAGTAAAAAGAAAAATACAATGATGACTACCGGACAGAGAAATTTGCAGGCTTGTGAG
159 A  P  S  S  L  D  W  R  K  K  G  V  V  T  P  I  K  N  Q  G
541 GCGCCTTCTAGCTTGGATTGGAGGAAGAAGGGAGTTGTCACTCCAATAAAGAACCAAGGA
179 N  C  G
601 AATTGTGgtaattttctataatttctccctcttatatatattacatatgtacatatatgc
661 atacttgatgtatatgtgaaggcataagacaccataaatgatatgacaggttttagataa
721 cttaaaatctaaagtaaaaagtactttgatgctaaagagaataaaaaaaaacgtaaatat
781 attcataggtataggttgaagtgatacaacatacttgagtttaacatatgatttaatcgt
841 tcataaaaaataattgaaagaaaagaaaaaaaggcagatgcccttttattattcataaac
```

901 tggctttcctgttattattcacttaccaagacgtaatcctagttaacattaggataataa

182 S

961 tgtgggaaattatcttcattttggtgtacttcaactagattgatatttgtttacagGAAG

183 C W S F S V T G A I E G I N A I V T G D

1021 TTGTTGGTCATTCTCTGTAACTGGCGCCATAGAAGGAATAAACGCCATAGTTACTGGTGA

203 L I S L S E Q E L V D C D T T N F G C D

1081 CCTAATTAGCCTTTCAGAACAAGAACTTGTAGATTGTGATACCACCAATTTCGGATGTGA

223 G G Y M D Y G F E W V I N N G G I D T E

1141 TGGAGGCTATATGGACTACGGTTTTGAATGGGTTATTAACAACGGTGGGATTGACACAGA

243 A N Y P Y T G V N G T C N I T K

1201 AGCGAATTATCCATATACTGGTGTAAATGGGACCTGCAATATCACCAAGgtttctttctt

1261 atctaaaatcttatttacccatatacccaatgcgaacatgatttaaaaccttaatctgtt

259 E E M K I V T V D

1321 gtgtgtctcattaatgatctttttgtttgatcagGAGGAAATGAAAATTGTTACCGTTGA

268 G Y E D V A K S D S A L F C A T A Q Q P

1381 TGGGTACGAGGATGTAGCTAAATCAGACAGTGCCCTGTTCTGTGCTACAGCTCAGCAACC

288 V S V G M D G S A E D F Q L Y T S

1441 TGTTTCTGTGGGTATGGATGGTTCTGCAGAAGATTTTCAATTGTATACAAGTgtaagtct

1501 ttttgttcgtttcatgagtacaattaaaaagataattactatagaagtgtttgataaaat

305 G I Y D G N C S S N P D D

1561 ggaaatgatgtgatatgatcagGGGATTTATGATGGTAATTGCTCCAGTAATCCAGACGA

318 I D H A V L I I G Y G S E G D E D Y W I

1621 CATTGATCATGCCGTTTTGATCATTGGGTATGGATCTGAAGGTGATGAAGATTATTGGAT

338 V K N S W G T D W G I D G Y F Y L R R N

1681 AGTGAAAAATTCGTGGGGTACTGACTGGGGTATCGATGGATACTTTTATTTAAGAAGGAA

358 T D L T Y G V C A V N A M A S Y P T K Q

1741 TACTGATTTAACATATGGCGTTTGTGCTGTTAATGCAATGGCTTCTTATCCTACTAAACA

378 S S A P S P T S P P S P L S P P P P P S

1801 ATCCTCTGCTCCATCTCCAACAAGTCCTCCTTCACCACTATCACCTCCTCCACCACCATC

398 L P P P P P P T P V P P P P P P S P S E

1861 ACTACCACCACCACCTCCTCCGACACCAGTGCCTCCACCTCCTCCTCCTTCACCAAGTGA

418 C G D F S Y C P S G E T C C C F L E I F

1921 ATGTGGAGATTTCTCTTACTGTCCAAGCGGTGAAACATGTTGTTGCTTTCTTGAAATCTT

438 D I C W A Y S C C P F E N A V C C P D S

1981 TGATATCTGTTGGGCATATAGTTGCTGTCCTTTTGAGAATGCTGTATGCTGCCCTGATTC

458 L D C C P S D Y P I C D V V E G L C L Q

2041 ATTAGATTGCTGCCCTAGTGATTACCCTATTTGTGATGTTGTGGAAGGCCTCTGCCTCCA

478

2101 Ggtaagaatcgtcaatttaatactccccatttcacatatataagtcggtttagagtaatt

2161 ttattgtctgtaaatataagtagtttttaaatattgtcaattcaaacagtagcattaaat

478 N P G D Y L G V

2221 acaccttagttaattgtttattgaaattgtgtaacagAACCCAGGAGATTACTTGGGAGT

486 A S R K R H M A K H K L P W S K K E Q S

2281 TGCTTCCAGGAAGCGGCATATGGCTAAGCACAAGCTTCCCTGGAGTAAAAAAGAGCAAAG

506 K R Q T Y Q P L V W K R N P F A A I R *

2341 CAAACGACAGACTTACCAACCTCTCGTGTGGAAGAGGAACCCTTTCGCTGCAATTCGC**TG**

2401 **A**aaaataatgtgattttcttattaatttaaagttgtttcattgcaactttttgaatgatc

2461 ctattacaaaaaaaacttatgttttataaatattattaggggaaaatttgcaatgtgtgt

2521 tccatttggattatggtttttttgattttgaattaaagattggtttcccttattatatatg

2581 tcttgcagctttttgcaagccaaaaattcagtattgttgtcaaggccttgtaataaacct

2641 tactaaatgggtttttaattaaaataaacaaatcatttttccatttgaaa

Supplementary File S11. The gene model for *JcTHI1*. The coding region is marked with uppercase letters, above which are its deduced amino acids. The transcribed untranslated regions, including 5’ UTR, intron and 3’ UTR sequences, are marked with lowercase letters. The start and stop codons are marked with bold letters.

1 ttcccccagaaaaaatggacagcaaagaagacatgaatgaaacaagaagtccaactgaaa

61 taacttgtgcaaaagtatgatctacgtaaatgataataactgcattccgtaagcaaccca

121 tatatcattcctgggatgtactaaaattgtcatttataagctcccattctccatagacat

1 M E S H I N L K K A G L I L I S L

181 cgtcagtagcc**ATG**GAGTCGCATATTAATTTAAAGAAAGCTGGCTTGATTCTTATATCTC

18 C I L L I H T M A D L E I H S L P R Y N

241 TCTGCATTCTATTGATACATACAATGGCAGATTTAGAAATCCATAGTCTGCCAAGATATA

38 P K A M R K R Y D R W L K H H G R K Y H

301 ACCCCAAAGCCATGAGAAAGAGGTATGACAGGTGGCTGAAACATCATGGTCGAAAATATC

58 N K D E Y Y L R F G I Y Q S N I Q F I D

361 ATAACAAAGACGAATATTATCTGCGGTTTGGAATATATCAATCAAACATTCAGTTCATTG

78 Y I N A Q K L P Y E L R D N K F A D L T

421 ACTACATCAATGCTCAGAAATTACCATACGAACTCAGAGATAACAAGTTTGCAGACTTGA

98 N D E F K S I Y L G F Q A N K H G R K K

481 CTAATGATGAGTTTAAATCTATCTACCTGGGTTTTCAGGCTAATAAACACGGTAGAAAGA

118 P S H E H G N S S D L P T S V D W I K E

541 AGCCTAGCCATGAGCATGGAAACTCCTCAGACTTGCCAACCTCAGTAGATTGGATAAAGG

138 G A V T P V K D Q G Q C G

601 AAGGCGCTGTAACCCCGGTTAAAGATCAAGGTCAATGTGgtatgaagctttgtttccctt

661 ttctttttcatttttttcttccctgagaaatttgagtccaagtgcactttttagaagtgc

721 ttttgttttatgccaattgtcaattaatcattttgttttattctaagaatttttggcatg

151 S C W A F S A V A A

781 acaagatctaaagtgacagttctatacagGAAGTTGTTGGGCATTCTCTGCAGTGGCAGC

161 V E G I N K I K T G K L V S L S E Q E L

841 TGTTGAAGGCATTAACAAAATCAAGACAGGAAAATTAGTATCTCTGTCAGAACAAGAGCT

181 V D C D T H N D S Q G C N G G F M E T A

901 TGTAGACTGTGATACTCACAATGATAGCCAGGGATGTAATGGTGGATTCATGGAAACAGC

201 F T Y I Q K Q G L S I E D D Y P Y E G K

961 GTTTACATACATTCAAAAGCAGGGGCTCTCCATTGAAGATGATTATCCCTATGAAGGAAA

221 D G N C H K N K N K N Q T V T I S G Y E

1021 AGATGGCAACTGCCACAAAAACAAAAATAAAAATCAGACAGTGACTATTAGTGGTTATGA

241 A V P A N D E K S L Q T A V A K Q P V S

1081 AGCAGTACCTGCCAATGATGAGAAAAGCCTACAAACTGCAGTTGCCAAGCAACCTGTATC

261 V A I D A G G F S F Q F Y S K G I F S G

1141 TGTTGCAATTGATGCCGGTGGTTTTTCATTTCAATTCTACTCAAAAGGAATCTTCAGTGG

281 F C G T Q L N H G V T A V G Y G E T D G

1201 CTTCTGTGGAACACAACTCAACCATGGAGTCACAGCAGTCGGCTACGGAGAAACTGATGG

301 K K Y W L V K N S W G T E W G D S G Y I

1261 TAAAAAGTACTGGCTCGTTAAAAATTCATGGGGTACAGAGTGGGGTGATTCTGGCTATAT

321 K L R R G S K G K K G I C G I A M E P S

1321 AAAACTGAGGCGTGGTTCCAAGGGCAAGAAAGGTATCTGTGGCATTGCCATGGAGCCTAG

341 Y P V K E Q E *

1381 CTACCCTGTCAAGGAACAGGAA**TAA**gaatcattttatatgtgcttggctgatcctggctt

1441 tagggtgacttgacaatctcttaacaagatgtcttccatattagtaattacatacaaatc

1501 caagtaaaagatattgctcatggaaattaatttaacaatagttaagctttgcaggaagta

1561 tggtagggaaaatgcatgccataagcataagaagatgggtaattccttcgtatcaaaatg

1621 gatattacatcattgattggagatccccaattcttgatataaaatgcttaacataacttt

1681 catctatctcatttgtcatttggcaaagcccgaaaaagaacccgcataccaatcacaagt

1741 ctttcgagcaactaaaaggtgttaatattttactttgttctggagtgaaaatgagactta

1801 aagtctgtaggtatatttaaggtttgaatgtatcagaatatggatgagcctcaagagcca

1861 tcattgcaacaaaaaaatttgcatggtattagcatcaattgactgattgaggtaaggctt

1921 tgcttactttgtttatataattgaaccaaattgtgtaagtacaacattaatcagttagca

1981 gaaatcagaattgtaaactaac

Supplementary File S12. The gene model for *JcSAG12H1*. The coding region is marked with uppercase letters, above which are its deduced amino acids. The transcribed untranslated regions, including 5’ UTR, intron and 3’ UTR sequences, are marked with lowercase letters. The start and stop codons are marked with bold letters.

1 M T K K Q S

1 caaagattccttctgtatagttatagatactctcttgatact**ATG**ACTAAAAAACAAAGC

7 K S I F L V F V L N I L T I W A T H T V

61 AAATCCATATTTCTGGTATTTGTGTTGAACATATTAACCATATGGGCTACACATACGGTT

27 C R P L N E E Y M L K R H E E W R A Q H

121 TGTCGTCCTCTTAACGAAGAATACATGTTAAAGAGGCATGAAGAATGGAGAGCCCAACAT

47 G R V Y K D T A E K Q K K Y L V F K D N

181 GGACGTGTCTACAAAGACACAGCAGAGAAACAGAAAAAATACCTGGTTTTTAAGGACAAC

67 L E R I E S F N N G V D R G Y K L G L N

241 CTTGAACGTATTGAATCCTTTAACAATGGTGTGGACCGTGGATACAAGCTAGGACTCAAC

87 K F A D L T D E E F Q A M H L G Y K S P

301 AAATTTGCAGACTTAACAGATGAGGAATTTCAGGCCATGCACCTTGGTTACAAGAGCCCA

107 P S K L M S T S K S R S F R Y R N V T S

361 CCCTCCAAATTAATGTCCACTTCAAAGTCCAGATCCTTTAGGTACAGAAATGTAACTTCT

127 V P T T I D W R K A G A V T P V K D Q G

421 GTGCCAACTACTATAGATTGGAGAAAGGCCGGTGCTGTGACCCCTGTCAAAGATCAAGGC

147 S C G

481 TCCTGCGgtgagtgctgatataatcttaatatgtaaggcagaagtagacctaggatttag

541 aaccttaacttgtcatactatgagacagactcaaccactgtcctattgctaaataaatac

601 acatgaaattgaacaatgattttgatcttaaaaatcatgctattgatgcataacagaaaa

661 gagcaatccccgcaccacaaatgaaaattcgtttctaataaatcattttttaatgtttaa

150 S C W A F S A V A A M E G I T K L

721 tgcatagGAAGTTGCTGGGCATTCTCAGCAGTGGCAGCAATGGAAGGGATCACAAAACTC

167 K T G N L I S L S E Q E L V D C D V A G

781 AAAACTGGCAATTTAATATCTTTATCAGAGCAAGAGCTCGTAGATTGCGACGTAGCAGGT

187 E D Y G C D G G F M D T A F Q Y I L K N

841 GAGGATTATGGTTGTGACGGAGGTTTCATGGACACTGCTTTCCAATATATCCTAAAAAAT

207 G G L T S E A N Y P Y Q G E D G I C S K

901 GGAGGTCTCACGAGTGAGGCTAATTACCCCTACCAAGGAGAAGATGGCATCTGCAGCAAG

227 K K T A T S M A K I T G Y E D V P S N S

961 AAGAAGACAGCAACTTCTATGGCTAAAATAACTGGATATGAAGATGTGCCATCTAACAGT

247 E K A L L Q A V A N Q P V S V A I D A S

1021 GAAAAGGCTCTCTTGCAAGCTGTGGCAAACCAACCAGTTTCTGTTGCTATTGATGCTAGT

267 G Y D F R F Y S S G V F K G D C P T D L

1081 GGGTATGACTTCAGATTTTACTCTTCTGGTGTTTTTAAAGGGGACTGTCCTACCGATCTC

287 N H A V T V I G Y G S S S D G T K Y W L

1141 AACCATGCTGTTACTGTAATTGGGTATGGTAGTAGCAGTGATGGTACTAAGTATTGGTTG

307 L K N S W G T G W G E N G Y M R M Q R E

1201 CTAAAGAATTCATGGGGCACCGGTTGGGGTGAGAATGGGTATATGAGGATGCAAAGGGAA

327 I S A N E G L C G I A M K A S Y P T A *

1261 ATTAGTGCAAACGAAGGCCTCTGTGGCATTGCCATGAAAGCTTCGTATCCAACTGCT**TGA**

1321 aattgaacaaggagaagcataatggcaataaatcctatttgatgtgttagtatcaggaaa

1381 tttataggctgtaaaatgttctgctatctgtgctcattctatctttcatgcagttgtata

1441 atttcagcaatatgtgtttactatccatgcataaaggttcatttattttagctatcaagt

1501 accaac

Supplementary File S13. The gene model for *JcSAG12H2*. The coding region is marked with uppercase letters, above which are its deduced amino acids. The transcribed untranslated regions, including 5' UTR, intron and 3' UTR sequences, are marked with lowercase letters. The start and stop codons are marked with bold letters.

```
  1                                          M  T  K  K  Q  S
  1 caaagattccttctttatagttatagatactctcttgatactATGACTAAAAAACAAAGC
  7 K  S  I  F  L  V  F  V  L  N  I  L  T  I  W  A  T  H  T  V
 61 AAATCCATATTTCTGGTATTTGTGTTGAACATATTAACCATATGGGCTACACATACGGTT
 27 C  R  P  L  N  E  E  Y  M  L  K  R  H  E  E  W  R  A  Q  H
121 TGTCGTCCTCTTAACGAAGAATACATGTTAAAGAGGCATGAAGAATGGAGAGCCCAACAT
 47 G  R  V  Y  K  D  T  A  E  K  Q  K  K  Y  L  V  F  K  D  N
181 GGACGTGTCTACAAAGACACAGCAGAGAAACAGAAAAAATACCTGGTTTTTAAGGACAAC
 67 L  E  R  I  E  S  F  N  N  G  V  D  R  G  Y  K  L  G  L  N
241 CTTGAACGTATTGAATCCTTTAACAATGGTGTGGACCGTGGATACAAGCTAGGACTCAAC
 87 K  F  A  D  L  T  D  E  E  F  R  A  M  H  L  G  Y  K  S  L
301 AAATTTGCAGACTTAACAGATGAGGAATTTCGGGCTATGCACCTTGGTTACAAGAGCCTA
107 P  S  K  L  M  A  T  S  K  S  R  S  F  R  Y  R  N  V  T  S
361 CCCTCCAAATTAATGGCCACTTCAAAGTCCAGATCCTTTAGGTACAGAAATGTAACTTCC
127 V  P  T  T  I  D  W  R  K  A  G  A  V  T  L  V  K  D  Q  G
421 GTGCCAACTACTATAGATTGGAGAAAGGCTGGTGCTGTGACCCTTGTCAAAGATCAAGGC
147 S  C  G
481 TCCTGCGgtaagtgctgatataatcttaatatataaggcagaagtagacctaggatttag
541 aaccttaacttgtcatactatgagacagactcaaccattgtcctattgctaaataaatac
601 acataaaaacaatgattttgatcttaaaaatcatgctattgatgcataacagaaaagagc
661 aatccccgtaccagaaatgaaaattcatttctaataaatcatttttaatgtttaatgca
150      C  C  W  A  F  S  A  V  A  A  M  E  G  I  T  K  L  K  T
721 tagGATGTTGCTGGGCATTCTCAGCAGTGGCAGCAATGGAAGGAATCACAAAACTTAAAA
169   G  K  L  I  S  L  S  E  Q  E  L  V  D  C  D  I  A  G  E  D
781 CTGGCAAGTTAATATCTTTATCAGAGCAAGAGCTCGTAGATTGTGACATAGCAGGTGAGG
189   Y  G  C  D  G  G  F  I  D  T  A  F  Q  Y  I  L  K  N  G  G
841 ATTATGGTTGTGACGGAGGTTTCATAGACACTGCTTTCCAATATATCCTAAAAAATGGAG
209   L  T  S  E  A  N  Y  P  Y  Q  G  E  D  G  I  C  S  K  K  K
```

901 GTCTCACGAGTGAGGCTAATTACCCCTACCAAGGAGAAGATGGCATCTGCAGCAAGAAGA

229 T A T S T A K I T G Y E D V P S N S E K

961 AGACAGCAACTTCTACGGCTAAGATAACTGGATATGAAGATGTGCCATCTAACAGTGAAA

249 A L L Q A V A N Q P V S V A I D A S G Y

1021 AGGCTCTCTTGCAAGCTGTGGCAAACCAACCAGTTTCTGTTGCTATTGATGCTAGTGGGT

269 D F R F Y S S G V F Q G D C T T Y L N H

1081 ATGACTTCAGATTTTACTCTTCTGGTGTCTTTCAAGGGGACTGTACTACCTATCTAAACC

289 A V T V I G Y G S S S Y G T K Y W L L K

1141 ATGCTGTTACTGTAATTGGGTATGGTAGTAGCAGTTATGGTACTAAGTATTGGTTGCTAA

309 N S W G T G W G E N G Y M R M Q R E I S

1201 AGAATTCATGGGGCACCGGTTGGGGTGAGAATGGGTATATGAGGATGCAAAGGGAAATTA

329 A N E G L C G I A M K A S Y P T A *

1261 GTGCAAATGAAGGCCTCTGTGGCATTGCCATGAAAGCTTCGTATCCAACTGCT**TGA**aatt

1321 gaacaggagaagcataatggcaataaatcctatttgatgtgttagtatcaggaaatttat

1381 aggctgcaaaatgttctgctatctgtgctcattctatctttcatgcagttgtataatttc

1441 agcaatatgtgtttaatatccatgtataaaggttcatttattttagctatcaagtaccaa

1501 t

Supplementary File S14. The gene model for *JcSAG12H7*. The coding region is marked with uppercase letters, above which are its deduced amino acids. The intron sequences are marked with lowercase letters. The start and stop codons are marked with bold letters.

```
  1 M  A  V  V  V  L  L  E  N  K  L  I  F  V  A  V  L  V  M  G
  1 ATGGCTGTAGTAGTACTACTTGAAAACAAACTAATCTTTGTTGCAGTGTTAGTGATGGGG
 21 L  C  A  C  Q  G  W  S  R  S  L  Y  E  A  S  L  E  E  R  H
 61 TTGTGTGCTTGCCAAGGCTGGTCACGTTCCCTCTATGAAGCTTCGTTGGAGGAGAGGCAT
 41 E  I  W  M  A  Q  H  E  R  V  Y  K  D  A  T  E  K  E  M  R
121 GAAATTTGGATGGCCCAACATGAACGTGTTTATAAAGACGCGACAGAAAAGGAGATGCGC
 61 F  Q  I  F  R  E  N  V  K  F  I  E  S  F  N  K  A  G  N  K
181 TTTCAGATATTCAGGGAGAATGTGAAATTCATTGAATCTTTCAACAAGGCTGGGAATAAG
 81 S  Y  K  L  G  I  N  A  F  T  D  L  T  N  K  E  F  R  A  S
241 TCTTATAAGCTAGGGATTAATGCATTTACAGACCTCACTAATAAGGAATTTCGTGCATCG
101 R  N  G  Y  K  R  S  S  Y  S  S  S  S  G  T  N  S  F  K  Y
301 CGAAATGGATACAAGAGGTCTTCTTATTCAAGCTCATCAGGAACAAACTCATTCAAATAT
121 E  N  V  T  T  I  P  S  S  L  D  W  R  S  K  G  V  V  T  P
361 GAAAATGTCACTACAATTCCATCTAGCTTGGATTGGAGAAGCAAAGGAGTCGTTACACCC
141 I  K  D  Q  G  Q  C  G
421 ATCAAAGACCAAGGTCAATGTGgtaagtaacttggacttcatgtaaacaagtcagggttt
481 caaaatcctgatagtccatccctttaagaattgtgataaaagaaaaaaataggaaaagga
149                                                       C  C  W
541 aaaaagaaagaaacctcttaacattatatgaattgcatgcaaaaatgcagGATGTTGTTG
152  A  F  S  A  V  A  A  M  E  G  I  T  K  L  S  T  E  K  L  I
601 GGCATTCTCTGCGGTGGCAGCTATGGAAGGGATCACAAAACTCTCAACTGAGAAATTGAT
172  S  L  S  E  Q  E  L  I  D  C  D  T  S  G  I  D  Q  G  C  E
661 CTCCCTTTCTGAGCAAGAATTGATTGACTGTGATACAAGTGGAATAGACCAAGGCTGTGA
192  G  G  L  M  D  D  A  F  E  F  I  I  Q  N  N  G  L  T  T  E
721 GGGTGGTCTTATGGATGATGCCTTTGAATTCATTATTCAAAATAATGGCCTTACGACTGA
212  A  N  Y  P  Y  Q  A  E  D  G  T  C  N  T  E  K  A  A  N  H
781 AGCAAATTATCCTTACCAGGCAGAGGATGGAACTTGCAACACCGAGAAAGCAGCAAATCA
232  A  A  T  I  T  S  Y  E  D  V  P  E  N  N  E  E  A  L  R  M
841 TGCTGCCACAATCACAAGTTATGAAGATGTGCCAGAAAACAATGAGGAGGCCTTACGCAT
```

252 A V A N Q P V S V A I D A G E S A F Q H

901 GGCAGTGGCAAACCAACCAGTTTCTGTTGCCATTGATGCTGGTGAATCTGCCTTCCAACA

272 Y S S G I F T G D C G T E L D H G V T V

961 CTACTCGAGTGGAATATTTACTGGAGATTGTGGAACTGAATTAGACCACGGTGTCACTGT

292 V G Y G T S D D G T K Y W L V K N S W G

1021 AGTCGGTTATGGGACAAGTGATGATGGGACTAAGTATTGGTTAGTAAAGAACTCATGGGG

312 T S W G E D G Y I R M Q R D I D A K E G

1081 AACCAGCTGGGGTGAGGATGGATACATTAGAATGCAAAGAGACATTGATGCTAAAGAAGG

332 L C G I A M Q P S Y P T A *

1141 CCTATGTGGAATTGCCATGCAGCCTTCCTATCCAACTGCA**TAA**

Supplementary Fig. S1. Detailed information of 15 motifs identified in this study.

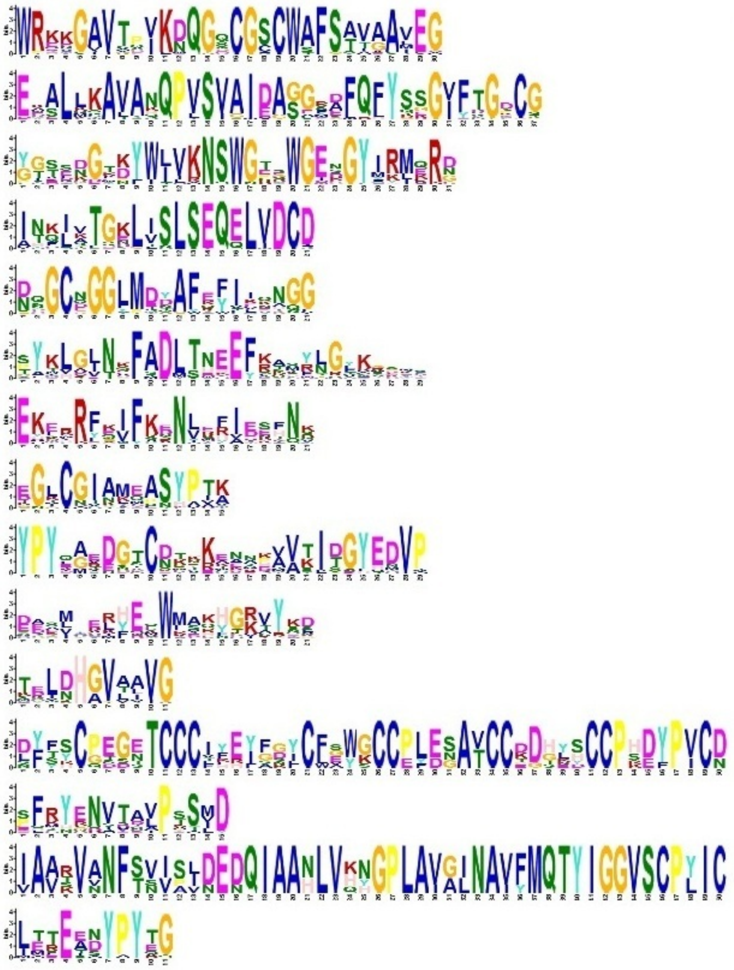

	Logo	E-value	Sites	Width
Motif 1.		1.3e-1107	49	30
Motif 2.		1.5e-881	36	37
Motif 3.		1.6e-875	44	31
Motif 4.		1.8e-638	44	21
Motif 5.		1.0e-565	49	21
Motif 6.		1.2e-597	47	29
Motif 7.		3.4e-445	47	21
Motif 8.		1.7e-342	48	15
Motif 9.		6.1e-390	28	29
Motif 10.		2.8e-273	39	21
Motif 11.		7.3e-234	48	11
Motif 12.		5.5e-194	8	50
Motif 13.		9.9e-141	26	15
Motif 14.		2.6e-110	5	50
Motif 15.		3.6e-062	17	11

Supplementary Fig. S2. Matched positions of 25 RcPLCP genes on physic nut chromosomes. The positions were based on the synteny analysis and only 8 matched chromosomes were shown. RcPLCP genes were shown behind that of physic nut.

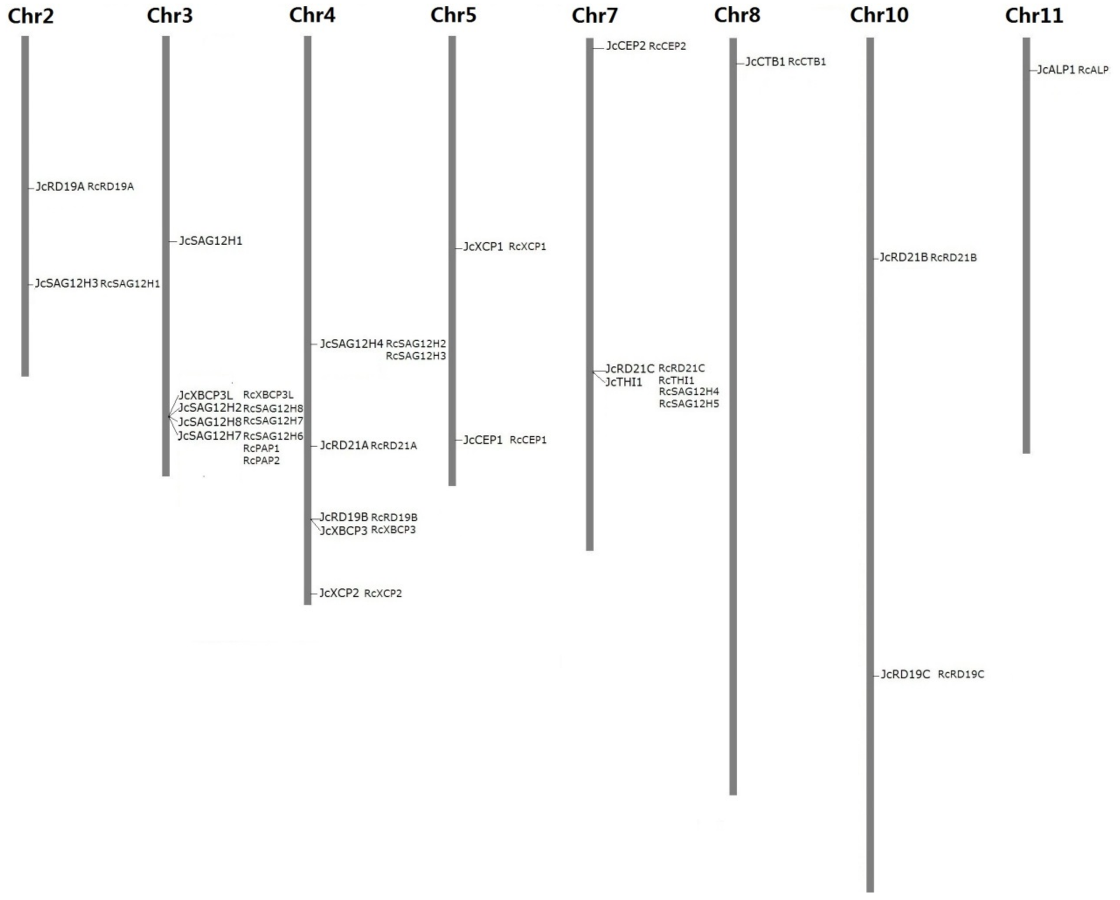

Supplementary Table S1. Detailed information of 31 PLCP genes in *Arabidopsis*.

Subfamily	Gene name	Other names	TAIR ID	Chr	Nucleotide length (bp, from start to stop codons)		Intron NO.	AS[1]	AA	MW (kDa)	*p* I	GRAVY	iPSORT[2]	Duplicate	Mode[3]
					CDS	Gene									
RD21	*AtRD21A*	RD21	AT1G47128	Chr1	1389	2471	4	-	462	50.97	5.26	-0.446	S		
	AtRD21B		AT5G43060	Chr5	1392	2334	4	-	463	51.20	5.86	-0.457	S	AT1G47128	Beta
	AtRD21C		AT3G19390	Chr3	1359	1745	4	-	452	49.31	5.60	-0.368	S		
	AtRDL1	CP1	AT4G36880	Chr4	1131	1489	3	-	376	41.64	7.54	-0.505	S	AT4G11310	Proximal
	AtRDL2		AT3G19400	Chr3	1089	1369	3	Yes	362	40.29	4.77	-0.322	S	AT3G19390	Tandem
	AtRDL3		AT3G43960	Chr3	1131	1507	3	-	376	41.54	5.08	-0.235	S	AT3G19390	Transposed
	AtRDL4	CP1	AT4G11310	Chr4	1095	1725	3	-	364	39.92	5.69	-0.326	S		
	AtRDL5	CP2	AT4G11320	Chr4	1116	1492	3	-	371	40.71	5.58	-0.349	S	AT4G11310	Tandem
	AtRDL6		AT4G23520	Chr4	1071	1763	3	-	356	39.60	5.65	-0.378	S	AT4G11310	Alpha
CEP	*AtCEP1*		AT5G50260	Chr5	1086	1258	2	-	361	40.71	7.15	-0.613	S		
	AtCEP2		AT3G48340	Chr3	1086	1336	2	-	361	40.37	5.99	-0.617	S	AT5G50260	Beta
	AtCEP3		AT3G48350	Chr3	1095	1619	2	-	364	40.97	5.87	-0.576	M	AT3G48340	Tandem
XCP	*AtXCP1*		AT4G35350	Chr4	1068	1347	3	Yes	355	39.62	5.60	-0.449	S	AT1G20850	Gamma
	AtXCP2		AT1G20850	Chr1	1071	1330	3	-	356	39.71	5.16	-0.478	S		
XBCP3	*AtXBCP3*		AT1G09850	Chr1	1314	2028	4	-	437	48.07	6.02	-0.309	S		
THI	*AtTHI1*		AT1G06260	Chr1	1032	1137	1	-	343	37.77	7.51	-0.225	S		
SAG12	*AtSAG12*		AT5G45890	Chr5	1041	1460	2	-	346	38.23	7.98	-0.321	S		
	AtPAP1		AT2G34080	Chr2	1038	1347	1	-	345	38.27	6.90	-0.350	S		
	AtPAP2		AT1G29090	Chr1	1068	1283	1	-	355	39.94	6.12	-0.363	S	AT1G29080	Tandem
	AtPAP3		AT1G29080	Chr1	1041	1181	1	-	346	38.79	5.74	-0.295	S	AT2G34080	Alpha
	AtPAP4		AT2G27420	Chr2	1047	1209	1	-	348	38.74	5.32	-0.351	C	AT2G34080	Transposed
	AtPAP5		AT3G49340	Chr3	1026	1231	1	-	341	37.69	4.56	-0.307	C	AT2G34080	Transposed
RD19	*AtRD19A*	RD19	AT4G39090	Chr4	1107	1501	2	-	368	40.42	6.75	-0.306	S		
	AtRD19B		AT2G21430	Chr2	1086	1338	2	-	361	39.82	7.08	-0.252	S	AT4G39090	Alpha
	AtRD19C		AT4G16190	Chr4	1122	1366	3	-	373	41.26	6.50	-0.252	S	AT4G39090	Gamma
	AtRD19D		AT3G54940	Chr3	1104	1726	3	-	367	40.13	6.79	-0.198	S		
ALP	*AtALP*	AALP, ALEU, SAG2	AT5G60360	Chr5	1077	2109	7	Yes	358	38.96	6.26	-0.186	S		
	AtALP2		AT3G45310	Chr3	1077	1770	7	Yes	358	39.54	6.22	-0.271	S	AT5G60360	Alpha
CTB	*AtCTB2*	CATHB2	AT1G02305	Chr1	1089	2059	10	-	362	40.03	6.45	-0.217	S		
	AtCTB1	CATHB1	AT1G02300	Chr1	1140	2089	9	-	379	42.26	6.74	-0.058	S	AT1G02305	Tandem
	AtCTB3	CATHB3	AT4G01610	Chr4	1080	2081	10	Yes	359	39.42	5.79	-0.235	S	AT1G02305	Alpha

[1] "Yes" represents genes containing alternative splicing isoforms; [2] "S, M and C" represent signal peptide, mitochondrial targeting peptide or chloroplast transit peptide, respectively, which was predicted by iPSORT; [3] duplicated modes were determined based on the study of Wang et al. (2013).

Supplementary Table S2. List of orthologous groups of the PCLP gene family based on the analysis of representative plant species.

Subfamily	OG	*R. communis*	*J. curcas*	*A. thaliana*	*M. esculenta*	*P. trichocarpa*	*C. papaya*	*T. cacao*	*V. vinifera*	*A. lyrata*	*B. oleracea*	*B. rapa*	*A. coerulea*	*O. sativa*	*A. trichopoda*	*S. moellendorffii*	*C. reinhardtii*
RD21				*AtRD21A*	Manes.18G041100					AL1G53290	Bol037029	Brara.J00562		LOC_Os04g57440			
	I	*RcRD21A*	*JcRD21A*		Manes.05G176900	Potri.014G024100	evm.TU.supercontig_21.158	Thecc1EG033775	GSVIVG01009440001		Bol045183	Brara.E01034	Aqcoe5G143300	LOC_Os04g57490	evm_27.TU.AmTr_v1.0_scaffold00051.91	270344	Cre09.g407700
				AtRD21B						AL8G17690	Bol015225	Brara.F03825					
	II	*RcRD21B*	*JcRD21B*	-	Manes.10G077200	Potri.001G302100<br>Potri.009G098100	evm.TU.supercontig_200.16	Thecc1EG007113	GSVIVG01003179001	-	-	-	-	-	-	-	-
	III	*RcRD21C*	*JcRD21C*	*AtRDL1*	Manes.13G119500	Potri.005G141600<br>Potri.007G047600	evm.TU.supercontig_55.102	Thecc1EG000683	GSVIVG01022063001	AL7G13640<br>-	Bol032703<br>-	Brara.A00157<br>Brara.H01714					
		-	-	*AtRD21C*	-	-	-	-	-	AL3G32800	Bol018095	Brara.E02264	-	-	-	-	-
	IV	-	-		-	-	-	-	-		Bol031006		-	-	-	-	-
		-	-	*AtRDL2*	-	-	-	-	-	AL3G32810	-	-	-	-	-	-	-
		-	-	*AtRDL3*	-	-	-	-	-	AL5G21990	Bol018097	Brara.E02263	-	-	-	-	-
		-	-	*AtRDL4*	-	-	-	-	-	AL6G45710	Bol012399	Brara.B02684	-	-	-	-	-
	V	-	-	*AtRDL5*	-	-	-	-	-	AL6G45720			-	-	-	-	-
		-	-	*AtRDL6*	-	-	-	-	-	AL7G29890	Bol014912	Brara.A01362	-	-	-	-	-
CEP	I			*AtCEP1*	Manes.06G113000	Potri.012G090900				AL8G24300	Bol015079	Brara.C02533					
		RcCEP1	*JcCEP1*	*AtCEP2*	Manes.14G056700	Potri.015G087400	evm.TU.supercontig_4.28	Thecc1EG014527	GSVIVG01007585001	AL5G27760	-	Brara.F01680	Aqcoe3G136400	LOC_Os11g14900	evm_27.TU.AmTr_v1.0_scaffold00049.113	-	-
				AtCEP3		Potri.015G087500				AL5G27750	-	Brara.F01682					
	II	*RcCEP2*	*JcCEP2*	-	Manes.12G019800	Potri.008G183100	evm.TU.supercontig_33.75	Thecc1EG010654	GSVIVG01000582001	-	-	-	-	-	-	-	-
XCP	I	*RcXCP1*	*JcXCP1*	*AtXCP1*	Manes.03G097200<br>Manes.15G113300	Potri.004G207600	evm.TU.supercontig_232.4	Thecc1EG034834	GSVIVG01023863001	AL7G15810	Bol029080	Brara.A00297	Aqcoe5G113800<br>Aqcoe6G164200	LOC_Os01g73980<br>LOC_Os05g01810	evm_27.TU.AmTr_v1.0_scaffold00022.170<br>evm_27.TU.AmTr_v1.0_scaffold00022.173	-	-
	II	*RcXCP2*	*JcXCP2*	*AtXCP2*	Manes.05G127000	Potri.002G005700<br>Potri.005G256000	evm.TU.supercontig_286.15	Thecc1EG034834	GSVIVG01013420001	AL1G33870	Bol007014	Brara.H02309<br>Brara.G01086					
XBCP3	I	*RcXBCP3*	*JcXBCP3*	*AtXBCP3*	Manes.18G019400	Potri.005G232900	evm.TU.supercontig_698.4	Thecc1EG034834	GSVIVG01009796001	AL1G20450	Bol036791	Brara.F00644	Aqcoe5G182200	LOC_Os05g43230	evm_27.TU.AmTr_v1.0_scaffold00002.349		
	II	*RcXBCP3L*	*JcXBCP3L*	-	Manes.16G040500	Potri.004G057700<br>Potri.011G066800<br>Potri.011G066900	evm.TU.supercontig_7.76	Thecc1EG029686	GSVIVG01021223001	-	-	-	Aqcoe4G042400	-	-	-	-
THI	I	*RcTHI1*	*JcTHI1*	*AtTHI1*	Manes.13G139700	-	evm.TU.supercontig_209.7	Thecc1EG003574	GSVIVG01020657001	AL1G16260	Bol023341	Brara.H02966	Aqcoe1G243200	-	-	-	-
SAG12	Ia	-	*JcSAG12H1*<br>*JcSAG12H2*	*AtSAG12*	Manes.16G038400	Potri.004G055900	-	-	-	AL8G12660	Bol006318	Brara.B02856	Aqcoe1G352500<br>Aqcoe3G042700<br>Aqcoe3G042800<br>Aqcoe4G145200	LOC_Os04g12660<br>LOC_Os04g13090<br>LOC_Os04g13140<br>LOC_Os12g17540	evm_27.TU.AmTr_v1.0_scaffold00061.270<br>evm_27.TU.AmTr_v1.0_scaffold00061.271<br>evm_27.TU.AmTr_v1.0_scaffold00061.272<br>evm_27.TU.AmTr_v1.0_scaffold00061.273	-	-
	Ib	*RcSAG12H1*	*JcSAG12H3*	-	-	-	-	-	-	-	-	-	-	-	-	-	-
		RcSAG12H2	*JcSAG12H4*	-	Manes.01G149300	Potri.005G088600	evm.TU.supercontig_103.42	Thecc1EG001693	GSVIVG01020693001	-	-	-	-	-	-	-	-
		RcSAG12H3	-	-	Manes.01G149400	Potri.005G089100	evm.TU.supercontig_103.43	Thecc1EG001811	GSVIVG01020694001	-	-	-	-	-	-	-	-
		-	*JcSAG12H5*	-	-	Potri.007G075300	evm.TU.supercontig_103.44	Thecc1EG001814	GSVIVG01020696001	-	-	-	-	-	-	-	-
		-	*JcSAG12H6*	-	-	Potri.007G075900	-	-	GSVIVG01020697001	-	-	-	-	-	-	-	-
		-	-	-	-	Potri.007G076000	-	-	-	-	-	-	-	-	-	-	-
		-	-	-	-	Potri.007G076100	-	-	-	-	-	-	-	-	-	-	-
	Ic	*RcSAG12H4*<br>*RcSAG12H5*	-	-	Manes.13G139200	Potri.013G126100<br>Potri.013G118200	evm.model.supercontig_209.14<br>evm.model.supercontig_209.17	-	GSVIVG01021194001	-	-	-	-	-	-	-	-
		RcSAG12H6	*JcSAG12H7*	-	Manes.15G123400	Potri.011G064900	-	Thecc1EG029709	GSVIVG01021195001								
	Id	*RcSAG12H7*	-	-	Manes.16G037500	-	-	-	-	-	-	-	-	-	-	-	-
		RcSAG12H8	*JcSAG12H8*	-	Manes.16G037600	-	-	-	-	-	-	-	-	-	-	-	-
		-	-	-	Manes.16G037700	-	-	-	-	-	-	-	-	-	-	-	-
		-	-	-	Manes.17G054300	-	-	-	-	-	-	-	-	-	-	-	-
		-	-	-	Manes.17G054400	-	-	-	-	-	-	-	-	-	-	-	-
	II	*RcPAP1*	-	*AtPAP1*	Manes.17G055400	-	-	-	-	AL4G29990	-	Brara.D02074	-	-	-	-	-
		RcPAP2	-		-	-	-	-	-			Brara.E01011	-	-	-	-	-
		RcPAP3	-	*AtPAP2*	-	-	-	-	-	AL1G42810	Bol041861	-	-	-	-	-	-
		-	-	*AtPAP3*	-	-	-	-	-	AL1G42800	-	Brara.I02923	-	-	-	-	-
		-	-	*AtPAP4*	-	-	-	-	-	AL4G21360	-	Brara.A02184	-	-	-	-	-
		-	-	-	-	-	-	-	-	-	-	Brara.F01608	-	-	-	-	-
		-	-	-	-	-	-	-	-	-	-	Brara.F02067	-	-	-	-	-
		-	-	*AtPAP5*	-	-	-	-	-	AL5G29100	Bol021939		-	-	-	-	-
RD19	I	*RcRD19A*	*JcRD19A*	*AtRD19A*<br>*AtRD19B*	Manes.04G085600<br>Manes.11G087200	Potri.004G160300<br>Potri.009G121300	evm.TU.supercontig_3257.1	Thecc1EG006819	GSVIVG01024210001	AL7G11020<br>AL4G10610	-<br>Bol006722	Brara.A00027<br>Brara.D01264	Aqcoe5G181900	LOC_Os02g27030<br>LOC_Os04g24600	evm_27.TU.AmTr_v1.0_scaffold00001.80	266583	-
	II	*RcRD19B*	*JcRD19B*	*AtRD19C*	Manes.18G018400	Potri.005G234000	evm.TU.supercontig_150.51	Thecc1EG034140	GSVIVG01009807001	AL7G38900	Bol037199	Brara.A01927	-	-	-	-	-
		-	-	-	-	-	-	-	GSVIVG01009809001	-	-	Brara.C04487	-	-	-	-	-
									GSVIVG01009810001								
		-	-	-	-	-	-	-	GSVIVG01018882001	-	-	-	-	-	-	-	-
	III	*RcRD19C*	*JcRD19C*	*AtRD19D*	Manes.10G050900	Potri.010G228400	evm.TU.supercontig_53.142	Thecc1EG043315	GSVIVG01016190001	AL5G36050	Bol045388	Brara.I03759	Aqcoe1G131100	LOC_Os07g29760	evm_27.TU.AmTr_v1.0_scaffold00022.391	-	-
ALP	I	*RcALP1*	*JcALP1*	*AtALP*	Manes.16G095100	Potri.006G141700	evm.TU.supercontig_28.31	Thecc1EG037524	GSVIVG01015078001	AL8G36970	Bol008941	Brara.J01397	Aqcoe4G147600	LOC_Os09g27030	evm_27.TU.AmTr_v1.0_scaffold00024.285	439207	-
		-	-	*AtALP2*	Manes.03G040700	-	-	-	-	-	-	Brara.J01397	-	-	-	-	-
CTB	I			*AtCTB2*	Manes.05G006300	Potri.002G184300				AL1G11640	Bol004850	Brara.H03120					Cre02.g119900
		RcCTB1	*JcCTB1*		Manes.01G236900	Potri.014G109800	evm.TU.supercontig_57.55	Thecc1EG004987	GSVIVG01028272001	-	Bol007448	-	Aqcoe2G258000	LOC_Os05g24550	evm_27.TU.AmTr_v1.0_scaffold00045.14	143272	Cre12.g498850
				AtCTB1						AL1G11630	-	-					Cre16.g659750
				AtCTB3						AL6G51730	Bol011427	Brara.I00106					

“OG” represents “orthologous group”. “-” represents “Not Detected”.
